# Effects of steam processing conditions on the aroma and “dryness-like” effect of *Citri Sarcodactylis fructus*(FoShou tea)

**DOI:** 10.1016/j.fochx.2026.103590

**Published:** 2026-02-01

**Authors:** Xinhang Cai, Lishan Chen, Wangjun Li, Hu Wang, Yue Sun, Xingyang Xue, Shumei Wang, Menghua Wu, Jiang Meng

**Affiliations:** a Department of Gastroenterology, The First Affiliated Hospital of Guangdong Pharmaceutical University, Guangzhou, Guangdong, China; b College of Traditional Chinese Medicine, Guangdong Pharmaceutical University/State Administration of Traditional Chinese Medicine Key Laboratory of Digital Quality Evaluation of Chinese Medicinal Materials/Guangdong Provincial General Colleges and Universities Engineering and Technology Research Center for Quality of Chinese Medicinal Materials, Guangzhou, China; c Affiliated Cancer Hospital of Guangzhou Medical University, Guangzhou, China; d College of Pharmacy, Jinan University, Guangzhou, China

**Keywords:** *Citri Sarcodactylis fructus*, UHPLC-MS/MS, HS-GC–MS, Food composition, “Dryness-like” effect, Electronic nose

## Abstract

This study systematically explored the impact of steaming processing on *Citri Sarcodactylis Fructus*, focusing on changes in aroma, “dryness-like” properties, and the underlying chemical mechanisms. By employing HS-GC–MS, UHPLC-MS, electronic nose analysis, and animal experiments, we found that steaming triggered distinct changes in flavor substances: fresh, citrus-like volatiles diminished, while woody and bitter compounds increased, leading to a more intense aroma. It also drove the transformation of active components: flavonoid glycosides were converted to aglycones, with coumarins and limonoids were rearranged. These changes alleviated “dryness-like” effects: steamed CSF reduced rat water intake and blood viscosity (*p* < 0.05), normalized the aquaporins expression. Molecular and cellular assays linked this to stronger aglycone-AQP binding and weaker glycoside-induced AQP5 inhibition, which directly linked steaming to reduced dryness. This research clarified how steaming enhances aroma complexity and reduces “dryness-like” properties through targeted chemical transformations, providing a scientific basis for optimizing CSF processing via steaming.

## Highlights

• ‘Foshou’ tea results in stronger total aroma after steaming processing.
• Reduced ‘dryness’-like side effect of steamed ‘Foshou’ tea was evidenced on rats.
• ‘Dryness’-like effects of ‘Foshou’ tea in vivo was modulated through AQPs.
• The‘dryness’-like side effect of flavonoid glycosides is stronger than that of flavonoid aglycones.

## 1 Introduction

*Citri Sarcodactylis Fructus* (CSF, *Citrus medica* L. var. *Sarcodactylis* Swingle) is obtained by drying mature fingered citron. Like other citrus fruits, it is rich in flavonoids, polysaccharides, and other components. As a fruit product that is both edible and medicinal, CSF was regarded as a traditional functional tea drink around Asia. Therefore, in addition to its aroma characteristics as a food, its functional properties have also attracted much attention, such as anti-inflammatory effects and blood pressure-lowering activities, which make it still very popular as medicine and healthy tea beverages (Xu et al., 2024). Moreover, CSF tea was reported to possess the effects of drying dampness and resolving phlegm, as well as alleviating gastrointestinal motility disorders (Chen et al., 2025; Deng et al., 2025), further expanding its application value in traditional medicinal and health-promoting contexts.

Currently, two types of CSF tea were available: directly dried and sliced CSF tea (DCSF) and steamed CSF tea (SCSF), with the core difference being whether steaming was performed. Long-term consumption experience confirmd that steaming was key here. It significantly reduced CSF's irritancy and side effects, and mitigating the “dryness-like” effect stood as a primary, pivotal outcome of this steaming process. The “dryness-like” effect was usually characterized by phenomena such as thirst, constipation, and dry skin, which can cause discomfort for some consumers (Lan et al., 2025). However, there were relatively few comparative studies on these two types of CSF tea products. For example, a study compared the chemical compositions and gastroprotective effects of DCSF and SCSF. 42 active components were identified in both types of tea, with the same composition but significant differences in relative contents. In terms of gastroprotective effects, both DCSF and SCSF could alleviate gastric mucosal injury through antioxidant and anti-inflammatory activities, and SCSF showed better efficacy (Zhu et al., 2020). However, there were no reports on the overall chemical component migration and how this migration was related to the aroma and “dryness-like” effect changes of the two products. This posed a challenge for currently establishing a reference for balancing consumer sensory perception and functional requirements. In current research on tea processing, various treatment methods have been explored, including steaming, stir-frying, and cold extraction (Zhao et al., 2025). Steaming is another important processing method that played a crucial role in tea manufacturing: it can inactivate enzymes, preserve certain bioactive compounds, and influence the color and taste of tea (Liu et al., 2023). However, although some of these treatment methods have been investigated, the specific changes brought by steaming to CSF tea, such as its effects on nutritional value, bioactive compounds, activity, and in vitro health-promoting properties, remained poorly studied in detail.

Aroma, a pivotal sensory attribute of CSF, was tightly linked to its volatile components. The profile of volatile compounds directly shaped the distinctive fragrance of CSF, which significantly influenced consumer preference and selection, since aroma serves as the primary sensory cue for evaluating CSF's quality and palatability (Saintila et al., 2025). Meanwhile, a major challenge in CSF consumption lay in its pronounced “dryness-like” effect, characterized by thirst, disrupted body fluid balance, and altered whole-blood viscosity (Zhang et al., 2024). This drying effect was strongly associated with aquaporins (AQPs), particularly AQP2, AQP3, and AQP5, which regulated water metabolism in the kidney, colon, and submandibular gland, respectively (He & Yang, 2019; Ikarashi et al., 2019). Evidence indicated that DCSF-induced dryness was mediated through the modulation of these AQPs, leading to impaired water transport and subsequent dryness-related symptoms (Liu et al., 2023). To investigate this functional aspect, active components were prioritized because CSF is typically consumed orally; active components, such as flavonoids, remain stable during digestion and absorption, which allowed them to exert sustained effects on AQP regulation and water metabolism (Li et al., 2021). However, the specific mechanisms through which steaming modulates the aroma and “dryness-like” effect of CSF by altering volatile and active components remained unclear.

This study aimed to address this critical knowledge gap through the integration of multiple complementary analytical and experimental approaches. Initially, Headspace gas chromatography–tandem mass spectrometry (HS-GC–MS) was employed to characterize dynamic changes in volatile compounds, with electronic nose analysis supporting findings by profiling aroma fingerprints and linking volatile alterations to sensory aroma variations. Subsequently, In vivo rat models were utilized to assess functional indicators (water intake, whole-blood viscosity, AQP2, AQP3, AQP5 expression). Further, Ultra-high-performance liquid chromatography–tandem mass spectrometry (UHPLC-MS/MS) was applied to quantify chemical transformations in key bioactive constituents (flavonoids, coumarins, limonoids), focusing on flavonoid glycoside conversion to aglycones (a process influencing biological activity) for exploring chemical changes' relevance to the “dryness-like” effect. Finally, In vitro cell assays were conducted to determine representative flavonoids' regulatory effects on AQP5 expression, with molecular docking simulations elucidating binding affinities and interactions among flavonoids and aquaporins to uncover the structural basis for aglycone-associated attenuated drying effect. The present study provided a preliminary groundwork for further exploring the migration mechanisms of chemical components and their potential roles in mediating the dryness property of DCSF and SCSF.

## 2 Materials and methods

### 2.1 Samples and chemicals

Ten independent batches each of DCSF and SCSF were sourced from various suppliers in Guangdong, China. All samples were authenticated by Professor Jizhu Liu from Guangdong Pharmaceutical University as *Citri Sarcodactylis Fructus*. Both DCSF and SCSF were prepared in accordance with the 1984 edition of the Guangdong Provincial Processing Standards for Traditional Chinese Medicines. Fresh CSF was cleaned to remove impurities and cut into slices, then directly dried at 50 °C to obtain DCSF; DCSF was cleaned to remove impurities and then steamed for 3 h, and subsequently dried at 50 °C to obtain SCSF. Samples were deposited in the Herbarium of Guangdong Pharmaceutical University. Detailed experimental sample information and experimental materials are provided in Supplementary Tables S1 and S2.

### 2.2 Sample preparation

A total of 1.5 kg of DCSF, after being soaked in 95% ethanol (1:8, *w*/*v*) for 2 h, were extracted twice by 95% ethanol reflux (3 h and 1.5 h). The two extracts were combined, filtered, and concentrated under reduced pressure to recover ethanol. Subsequently, ultrapure water was added to fully dissolve the resulting extract. The preparation of SCSF was the same as that of DCSF for animal experiments.

All samples were ground (through 24 mesh sieve), sealed, and stored at 4 °C for electronic nose and HS-GC–MS analyses. 1.0 g of powdered sample (through 100 mesh sieve), the powder was extracted with 10 mL of 50% (*v*/v) methanol by 45 min ultrasonication at 40 kHz and 150 W. After standing, the supernatant was filtered through a 0.22 μm filter, and stored at 4 °C prior to LC-MS/MS analysis.

### 2.3 LC-MS/MS and HS-GC–MS analysis

Chromatographic separation was achieved by a Waters ACQUITY UPLC BEH C18 column (2.1 × 100 mm, 1.7 μm) on an Ultimate 3000 UHPLC system (Thermo Fisher Scientific), with a flow rate of 0.2 mL/min and an injection volume of 2 μL. Mobile phases were acetonitrile and 0.1% formic acid water under a multi-step gradient (Gradient details in Supplementary). The column oven was set at 35 °C. Detection was performed using an Orbitrap Fusion Tribrid Mass Spectrometer (Thermo Fisher), equipped with an ESI source. Both positive and negative modes were employed; full scan ranges were 100–1500 *m*/*z* (MS) and 50–1500 m/z (MS/MS). Calibration and data acquisition were managed via Xcalibur 4.0.

Volatile profiling used an Agilent 7697 A Headspace Sampler and 8890-7000D GC–MS (Agilent, USA) with a DB-5MS column (30 m × 0.25 mm, 0.25 μm). Helium was the carrier gas. Injection volume was 1 μL, split 5:1 (Gradient details in Supplementary). The oven temperature program ranged from 60 °C to 250 °C under set ramps. Electron impact ionization (70 eV) and mass scan range m/z 20–550 were used. MassHunter software was utilized for acquisition and integration. For 10 samples each of DCSF and SCSF, LC-MS and HS-GC–MS analyses were conducted 3 parallel samples per original sample, each measured once.

LC-MS raw files were processed by Compound Discovery 3.1 for peak picking, alignment (mass window 5 ppm, RT 0.2 min). HS-GC–MS data were analyzed via MassHunter B.06.00 and peaks were identified using the NIST 14 library (similarity ≥80%). Principal component analysis (PCA), partial least squares regression (PLSR), and orthogonal partial least squares discriminant analysis (OPLS-DA) were performed in SIMCA-P 14.1. OPLS-DA model and PLSR model quality parameters were reported as R^2^X, R^2^Y, and Q^2^, the optimal number of latent variables was determined via 10-fold cross-validation, and 200 random permutation tests were used to evaluate model overfitting.

### 2.4 Electronic nose analysis

Volatile fingerprinting was performed using the PEN3 electronic nose (Airsense Analytics, Germany) with 10 metal oxide semiconductor (MOS) sensors targeting different volatiles. For each measurement, 1 g of sample was equilibrated at 25 °C for 30 min in a sealed beaker. Each sample was analyzed in triplicate with 150 s sampling and cleaning times and 300 mL/min air flow. Details of the sensors are provided in Supplementary Materials. For 10 samples each of DCSF and SCSF, Electronic nose analyses were conducted 3 parallel samples per original sample, each measured once.

### 2.5 Methodology validation

LC-MS/MS was validated for repeatability, stability, and precision using standard solutions and sample solutions. HS-GC–MS was validated for repeatability using sample powders. Electronic nose was validated for repeatability using sample powders. Precision (repeatability and stability) was assessed as RSD of peak areas over repeated injections and over 24 h.

### 2.6 Relative odor activity values (ROAVs) analysis

Odor activity value (*OAV*) is a measure of the minimum threshold at which humans can perceive a taste. However, when the samples contained a large number of flavor compounds, their relative percentage content was used instead of their absolute content. The relative odor activity values (*ROAVs*) were based on the compound with the highest *OAV* in the sample, which served as a core metric for characterizing the aroma contribution of each volatile component via relative ratios and establishing a direct correlation between component presence and sensory perception. The compound with the highest overall flavor contribution in the sample was selected and recorded as *ROAV*_stan_ = 100. It should be clarified that the compound contributing most to the overall flavor was the one with the highest OAV rather than the highest concentration. Volatile compounds with *ROAV* ≥ 1 were key odor-active compounds, while those with 0.1 ≤ *ROAV* < 1 exerted a modulatory effect on the odor of each tea. The *ROAV* of other volatile organic compounds (*VOCs*) in the sample was computed using the following equation (Miao et al., 2024):

(1) $$\text{ROAV}=\left(\frac{C_{A}}{T_{A}}\right)\times \left(\frac{T_{\max}}{C_{\max}}\right)\times 100$$

C_max_ refers to the relative content of the component, which makes the greatest contribution to the overall flavor; T_max_ is the corresponding odor threshold (μg/kg); C_A_ denotes the relative content of the target component; T_A_ is the odor threshold of the target component (μg/kg).

### 2.7 Animal experiment

Male Sprague-Dawley (SD) rats (8 weeks, 180–220 g) were purchased from Zhuhai BesTest (Certification No. SCXK(Yue)2020–0051, Zhuhai, China). All procedures involving animal experiments in this study were carried out according to the requirements of the International AAALAC, and approved by the Institutional Ethics Committee (IEC) of Guangdong Pharmaceutical University (Ethics Approval number: GDPULAC2023132).

**Fig. 1:**
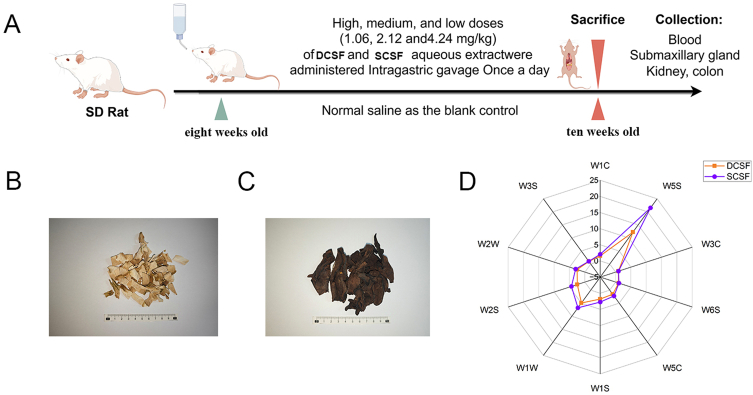
Appearance, electronic nose aroma simulation of two Citri Sarcodactylis Fructus with/without steaming processing. (A) Animal experimental procedure; (B) Appearance of directly dried and then sliced Citri Sarcodactylis Fructus(DCSF); (C) Appearance of steaming processing then dried and sliced Citri Sarcodactylis Fructus(SCSF); (D) Electronic nose detection results.

All rats were acclimated for 7 days, and housed under SPF conditions (20–26 °C, 40%–70% humidity, 12 h light/dark cycle) at Guangdong Pharmaceutical University. The detailed animal experiment procedure is shown in Fig. 1A. DCSF/SCSF extracts were prepared by ethanol reflux extraction, extracts were suspended in 0.5% CMC-Na for gavage. Rats were divided into control, low-dose, medium-dose, and high-dose groups (1.06, 2.12, 4.24 g/kg, respectively; *n* = 10/group). During the housing and administration period, rats' body weight, water intake, and activity were monitored. Rats were anesthetized with isoflurane, and blood as well as tissue samples (submandibular gland, colon, kidney) were collected for subsequent analysis.

### 2.8 Whole blood viscosity

Whole blood (4.5 mL) was collected with heparinized anticoagulation. Viscosity at low, medium, and high shear rates was measured at 37 °C using an HT-100 A Hemorheology Analyzer. Each sample was measured three times; results reported as average values. 6 samples were randomly selected from each group, and each sample was analyzed in triplicate.

### 2.9 Cell experiment

NCI-H292 human bronchial epithelial cells were cultured in RPMI-1640 medium with 10% FBS at 37 °C, 5% CO₂. Pure flavonoid compounds (hesperidin, neohesperidin, naringin, narirutin, rutin, hesperetin, naringenin, isovitexin, apigenin, quercetin) were dissolved, sterilized (0.22 μm), and applied to cells at a range of concentrations (2.5–500 μg/mL). Cell viability was assessed by MTT assay after 24 h incubation.

### 2.10 RT-qPCR

For animal RT-qPCR, total RNA was extracted from rat submandibular gland, colon, and kidney using the TRIzol method, quantified with an A260/A280 ratio of 1.8–2.1, reverse transcribed into cDNA, and then RT-qPCR was performed with *β*-actin as the reference gene to quantify the mRNA expression of aquaporins (AQP2, AQP3, AQP5), with PCR cycling conditions and primer sequences were provided in Supplementary Materials and relative gene expression was calculated using the 2^-ΔΔCt^ method. For cell RT-qPCR, 4 × 10^5^ cells/well were seeded in 6-well plates; after treatment, total RNA was extracted using the AG RNAex Pro Kit, quantified, and reversely transcribed to cDNA, followed by SYBR Green-based amplification was performed on a T100 Real-Time PCR System with *β*-actin as the housekeeping reference gene, and PCR cycling conditions and primer sequences were provided in Supplementary Materials. 6 samples were randomly selected from each group, and each sample was analyzed in triplicate.

### 2.11 Molecular docking simulation

AQP1 (PDB ID 7W7S), AQP2 (PDB ID 8J8Y), AQP4 (PDB ID 3GD8), AQP5 (PDB ID 3D9S) and a homology-modeled AQP3 (SwissModel, https://swissmodel.expasy.org/; GMQE = 0.7) were prepared for docking. (Hadidi & Kamali, 2021).Ligand structures were prepared using ChemDraw/Chem3D and processed in AutoDockTools 1.5.7; docking was performed using AutoDock Vina; binding energies were visualized using PyMOL 2.4.0.

### 2.12 Statistical analysis

In this study, data were presented as mean ± standard deviation (SD). Statistical analyses were carried out using GraphPad Prism 9.5.1. For multiple-group comparisons, one-way analysis of variance (ANOVA) was employed, and for comparisons between two groups, the *t*-test was used.

## 3 Results and discussion

### 3.1 Methodological validation results

The methodological validation results of LC-MS were presented in Tables S3. For precision assessment, the relative standard deviations (RSDs) of the peak areas of all components ranged from 1.16% to 4.87%; for repeatability evaluation, those of each component were between 0.7% and 3.67%; and for stability testing, those of all components varied from 0.85% to 3.61%. For HS-GC–MS, the selection of common peaks was illustrated in Fig. S1, with the methodological validation results were summarized in Table S3. The RSDs of the peak areas of all components ranged from 2.27% to 4.82%, and all values less than 5%. For the electronic nose, the methodological validation results were presented in Table S3. The RSDs of the sensor response values ranged from 0.19% to 2.86%, with all values less than 3%. Collectively, these results indicated that the established methods remained stable throughout the experiments.

### 3.2 Changes in aroma and appearance of CSF after steaming

Steaming induced changes in multiple aspects: it not only altered the appearance of the product but also affected its aroma and flavor. These characteristics precisely left a crucial first impression on consumers and influenced their consumption behavior (Bhavadharini et al., 2023). Meanwhile, steaming processing could inactivate microorganisms and enzymes, further extended the shelf life of the commodities (Fang et al., 2022).

The appearance of SCSF and DCSF was illustrated in Fig. 1B and Fig. 1C. DCSF exhibited a bright yellow hue, whereas SCSF displayed a dark brown coloration. This pronounced transformation was primarily attributed to the Maillard reaction during steaming. Specifically, the carbonyl groups of reducing sugars in DCSF condensed with the amino groups of amino acids to form unstable Schiff bases, which subsequently rearranged to yield Amadori products. These intermediates underwent further dehydration, fragmentation, and polymerization reactions, ultimately generated high-molecular-weight, brown pigments, which thereby deepened the food's color (El Hosry et al., 2025). The findings were consistent with previous reports on the effects of steaming on CSF (Zhu et al., 2020).

In addition to appearance, aroma and flavor were also important characteristics. Therefore, we adopted electronic nose technology to detect the odor of DCSF tea and SCSF tea. Previous investigations had utilized electronic nose systems to analyze the aroma profiles of DCSF and “Laoxianghuang,” albeit with different research emphases. For example, studies on “Laoxianghuang” had employed electronic nose sensors to assess the impact of various fermentation stages on aroma composition, revealed marked sensor response differences and significant shifted in volatile components after six months of fermentation (Yaqun et al., 2022). In contrast, research on CSF across different production regions had highlighted distinct aroma characteristics, such as prominent fruity, herbal, or floral notes, and had demonstrated correlations between aroma composition and factors like geographical origin, processing methods, or raw material variations (Xu et al., 2024).

Previous studies had revealed how processing methods or geographical origins influenced volatile components. However, the specific impact of steaming on the aroma of CSF tea, particularly the systematic comparison between DCSF tea and SCSF tea, remaind unaddressed. Therefore, our study employed electronic nose sensors to systematically compare their aroma profiles. As shown in Fig. 1D, sensors W6S, W3C, and W3S registered minimal responses; conversely, sensors W5S, W1W, and W2S exhibited strong responses, while W2W, W1C, W5C, and W1S displayed moderate but comparatively weaker signals. These results suggested that nitrogen oxides, sulfur-containing compounds, alcohols, as well as aldehydes and ketones constituted the primary aroma contributors in both DCSF and SCSF. Importantly, contrary to prevailing assumptions, our data revealed that the aroma responses for SCSF surpassed those of DCSF in several key sensors (W5S, W2W, W2S, W1W, W1S, and W5C), indicated an intensified aroma following steaming rather than a reduction.

The enhancement of aroma intensity following steaming paralleled our recent findings on Chenpi tea (Lan et al., 2025). It seemed to be a common change feature of citrus fruits. To verified this conjecture, we further analyzed the total volatile components through HS-GC–MS.

### 3.3 Analysis of content changes in volatile components

**Fig. 2:**
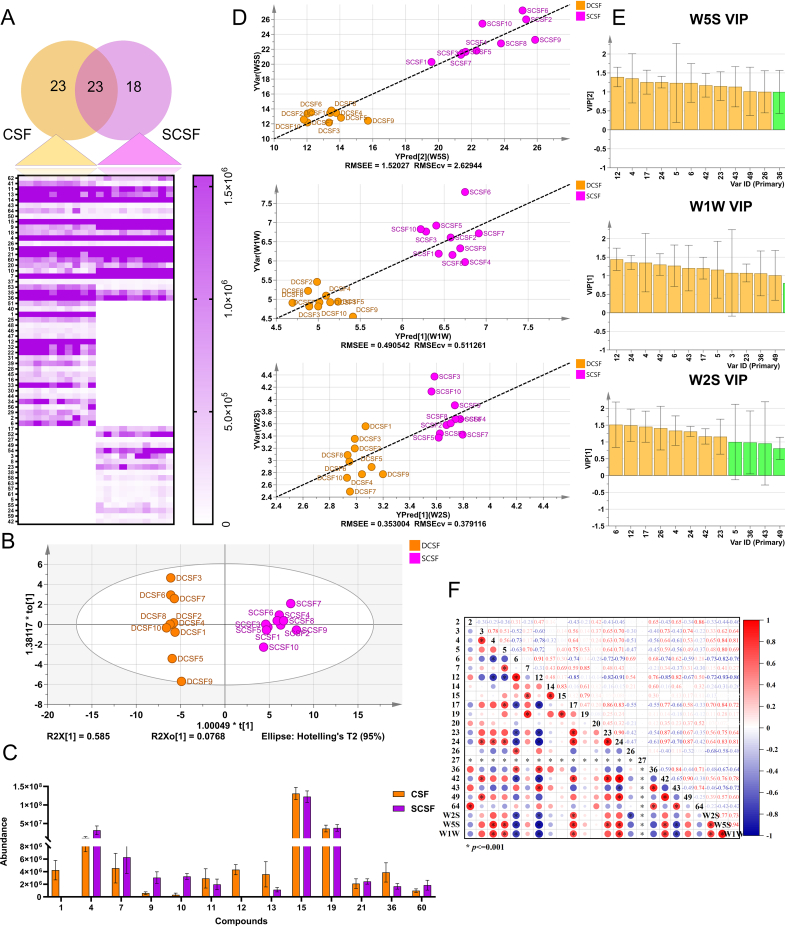
Analysis of volatile compounds identified by HS-GC–MS. (A) Venn diagram and heat map of volatile metabolites of DCSF vs SCSF; (B) PCA score scatter plot composed of all volatile metabolites of DCSF vs SCSF; (C) The top 10 components with the greatest changes in content between DCSF vs SCSF; (D) PLSR model established by using volatile metabolites as input and the data of electronic nose sensor as target; (E) VIP of the established PLSR model; (F) Pearson model Established with volatile metabolites (VIP > 1 in the PLSR model) as input and electronic nose sensor data as target.

A comprehensive identification and comparison of the volatile compounds in DCSF tea and SCSF tea were conducted. A total of 64 volatile compounds were identified (Table S4; total ion chromatograms (TICs) in Figs. S2 and S3). Venn analysis (Fig. 2A) revealed 23 compounds common to both DCSF and SCSF, with 23 and 18 unique to DCSF and SCSF, respectively, highlighted significant changes in volatile composition upon steaming. Principal component analysis (PCA) of the HS-GC–MS data provided additional insight consistent with the electronic nose findings. The DCSF and SCSF samples were distinctly separated along the first principal component, as shown in Fig. 2B, with all data points contained within the 95% confidence ellipse. This separation underscored the profound impact of steaming on the volatile profile, which significantly altered the volatile compound profile.

As illustrated in Fig. 2C and Table S4, among the top 10 components of DCSF sorted by relative content, *d*-limonene (compound 15) and 3-carene (compound 19) were the most abundant; in contrast, the top 10 components of SCSF by relative content were led by *d*-limonene and 3-furaldehyde. Among the top 10 components with the greatest relative content changes after steaming, 3-furaldehyde (compound 4) exhibited the largest increase, followed by α-Thujene (compound 7), 5-methylfuraldehyde (compound 9); With decreased contents which included α-terpinyl formate (compound 36), *β*-terpinene (compound 11), and α-terpinene (compound 13). Additionally, components such as (−)-*β*-pinene (compound 12) and 2-methyl-3-buten-2-ol (compound 1) were absent in SCSF.

**Fig. 3:**
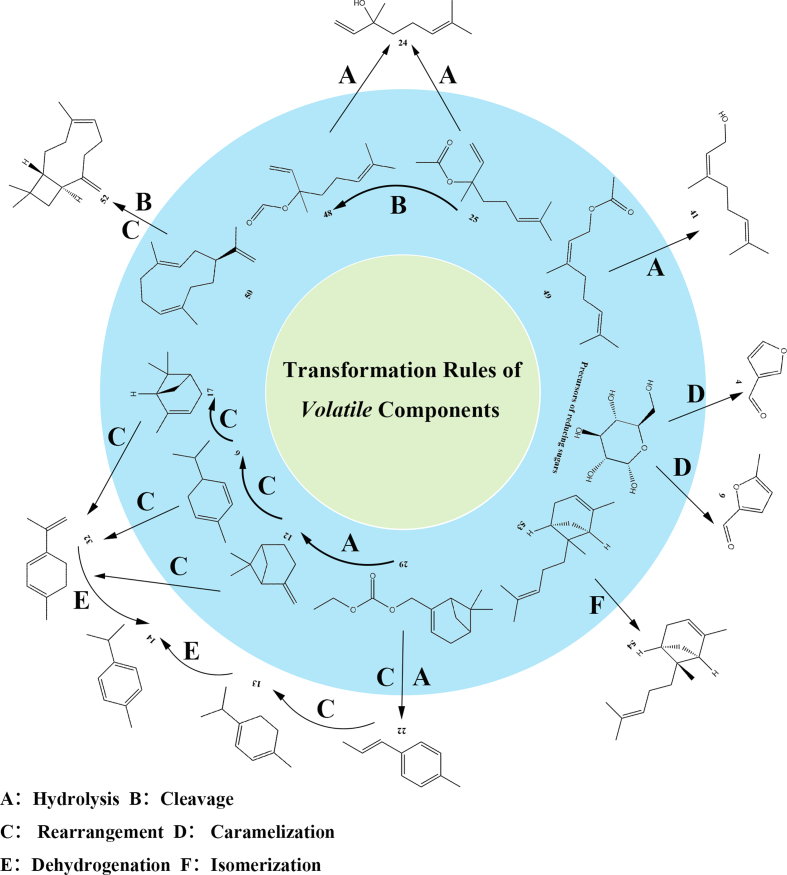
Possible conversion regularity of main volatile compounds metabolites between DCSF vs SCSF.

Based on the analysis of the aforementioned volatile components, the transformation rules of these components under steaming were identified, with the results presented in Fig. 3. The core conversion pathways could be categorized into six types of reactions: hydrolysis (A), cleavage (B), rearrangement (C), caramelization (D), dehydrogenation (E), and isomerization (F) (Deng et al., 2017). Hydrolysis (A) was the main consumption pathway for monoterpene esters, namely Linalyl acetate (component 25) and Neryl acetate (component 49). Under the action of high-temperature steam, these esters were rapidly decomposed into the corresponding alcohols: Linalool (component 24) and Nerol (component 41). Rearrangement and isomerization (C, F) of terpenes constituted another key mechanism. Unstable monoterpenes (−)-*β*-pinene underwent thermal rearrangement or cleavage to form *d*-limonene, which acted as a central intermediate for the subsequent transformation of complex terpene structures and could be further dehydrogenated to produce *p*-Cymene (component 14). Caramelization (D) dominated the flavor profile of steamed products. Reducing sugars underwent dehydration and depolymerization under thermal effects, generated 3-Furaldehyde and 5-methylfurfural (component 9) with sweet and roasted aromas. Meanwhile, dehydrogenation (E) promoted the conversion of cyclic terpenes to aromatic hydrocarbon derivatives, further enhanced the stability of the components. These reactions consumed some unstable components in DCSF and generated components of SCSF dominated by furfural and stable cyclic terpenes.

### 3.4 Analysis of aroma changes and correlation in volatile components

*E*-nose detection results revealed significant differences in odor response values between DCSF and SCSF. To clarify the alterations in aroma characteristics induced by steaming, *ROAVs* of volatile odor components were analyzed. As shown in Table 1, the *ROAVs* of partial volatile compounds were calculated to characterize their contribution intensity to the overall aroma profile.

**Table 1:** ROAV of differential volatile metabolites.

component	Identification	Order Threshold (μg/kg)	olfactory description	ROAV	
				DCSF	SCSF
2	Hexanal	33	Grass-like scent, fatty odor	0.6291	0.0000
3	(+)-2,3-butanediol	4500	Slightly sweet, no odor	0.0000	0.0020
4	3-Furaldehyde	77.3	Roasted scent, nutty odor	3.6387	5.0483
5	α-Angelica lactone	122	Creamy scent, coconut-like odor	0.0000	0.0078
6	α-Phellandrene	200	Citrusy scent, pine-like note	0.1900	0.0000
7	α-Thujene	20	Herbal scent, woody note	5.9222	3.8710
12	(−)-β-pinene	140	Grassy scent, pine-wood odor	0.8000	0.0000
14	p-Cymene	11.4	Aromatic, citrusy note	38.9562	15.5277
15	d-Limonene	34	Citrus scent, lemon-like odor	100.0000	44.4796
17	Toluene	330	Aromatic, sweet-spicy odor	0.0000	0.0143
19	3-Carene	770	Pine needle scent, resinous odor	1.2254	0.6104
20	*trans*-Linalool Oxide	5	Earthy scent, floral note	3.1108	1.6408
23	4-ethenyl-1,2-dimethylbenzene	34	Aromatic, mild sweet-spicy odor	0.0000	0.2923
24	Linalool	3.2	Floral scent, lavender-like odor	0.0000	1.6450
26	Nonanal	0.34	Orange scent, rose-like note	10.5275	3.2941
27	Maltol	162,000	Caramelized sweet scent, malt-like odor	0.0000	0.0000
36	*α*-terpinyl formate	60	Citrus scent, herbal note	1.6769	0.3439
42	Geraniol	0.014	Rose scent, lemon-like note	0.0000	100.0000
43	Thymol	0.93	Thyme-like scent, spicy odor	5.3183	0.7526
49	Neryl acetate	0.06	Orange blossom scent, sweet odor	0.0000	46.2000
64	Methyl palmitate	2000	No odor	0.0074	0.0015

Note: “-” represented that these thresholds were not reported; ROAV is the ratio of the concentration to the odor threshold.

The two teas differed significantly in key aroma components, with distinct changes in core contributors. DCSF had 9 key aroma components, featuring “fresh citrus and herbal-spicy notes”. *d*-limonene (reference component) provided signature citrus and lemon aromas (Vatankhah Lotfabadi et al., 2020), p-cymene boosted the fruity base with aromatic-citrus notes (Schreiner et al., 2020), and nonanal (component 26) enriched freshness with orange-rose aromas. Meanwhile, α-thujene and thymol added herbal layers via spicy notes (Ayu et al., 2025; Salehi et al., 2018), and hexanal (component 2), α-phellandrene (component 6), and (−)-*β*-pinene acted as modifiers, which lent a fresh herbal aroma and subtle oily note (Ghadiriasli et al., 2020). In contrast, SCSF also contained 9 key aroma components. The addition of linalool, geraniol (component 42), and neryl acetate (three highly active aroma components) shifted its aroma to “rich floral, citrusy sweetness” (de Groot, 2019). Geraniol, with the highest *ROAV* of 100 in SCSF, was selected as its reference component, imparted a signature rose and lemon-like sweet fragrance, while neryl acetate complemented it with orange blossom and sweet notes, and linalool contributed a lavender-like floral aroma (Elsharif & Buettner, 2018). Together, these three newly emerged components formed the sweet aromatic core. Among the retained original components, *d*-limonene maintained prominent citrus and lemon scents; 3-furaldehyde contributed distinct roasted and nutty aromas (Wang et al., 2024); *p*-cymene and nonanal retained aromatic-citrus and orange-rose notes respectively, though their aroma contributions weakened compared to DCSF. Additionally, *α*-thujene persisted as a key component, adding herbal-woody and earthy-floral layers. 4-ethenyl-1,2-dimethylbenzene remained as a modifier with mild aromatic and sweet-spicy notes. With the loss of herbal and pine needle notes from components like *α*-phellandrene and (−)-*β*-pinene, the overall aroma of SCSF was concentrated on rose, citrus, sweet, and roasted nutty scents.

After clarifying DCSF-SCSF differences in aroma components and *ROAV* characteristics, a correlation analysis was conducted to characterize the tea aroma-electronic nose sensor relationship. Using aroma component *ROAV* values and the three most responsive key sensors (W5S, W1W, W2S), PLSR and Pearson correlation analyses were conducted (Fig. 2D-F and Table 1); 200 permutation tests ruled out model overfitting (Fig. S4). Specifically, 3-furaldehyde, geraniol, and linalool were the key contributors to W5S (nitrogen oxides) and W2S (alcohols, aromatics) sensor responses, with extremely high VIP values and strong positive correlations with W5S which drove a significant rise in its SCSF response. In contrast, DCSF-exclusive (−)-*β*-pinene and hexanal showed significant negative correlations with these sensors (Wang et al., 2024), and their *ROAV* values dropped to 0 in SCSF, which reduced sensor responses. For W1W (sulfides, terpenoids), neryl acetate was strongly positively correlated and α-phellandrene strongly negatively correlated; this contrast meant W1W intuitively reflected the aroma reconstruction from herbal to orange blossom and sweet notes. In summary, steaming transformed the aroma profile from “citrus and herbal” to “rose, orange, and roasted notes”, which clarified the material basis for steaming-induced aroma changes—a trend consistent with recent findings (Lan et al., 2025).

### 3.5 Impact of steaming on CSF'S “dryness-like” effect

**Fig. 4:**
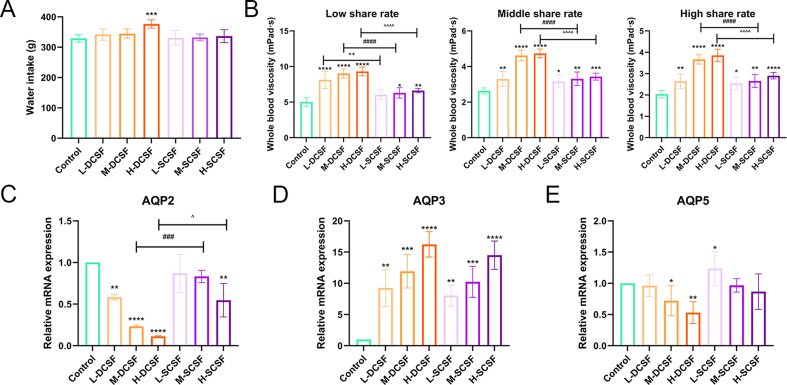
In vivo drying effect characterization of two Citri Sarcodactylis Fructus tea with/without steaming processing. (A) Effect of different doses of Citri Sarcodactylis Fructus tea on water intake in rats; (B) The whole blood viscosity of rats was characterized by high, medium and low shear rates; (C - E) Effects of Citri Sarcodactylis Fructus tea on mRNA expression levels of AQP5 in submandibular gland, AQP3 in colon and AQP2 in kidney in rats. *p < 0.05, ** *p* < 0.01, *** *p* < 0.001, **** *p* < 0.0001 vs Blank control group. + *p* < 0.05, ++ *p* < 0.01, +++ *p* < 0.001, ++++ *p* < 0.0001 L-DCSF group. vs L-SCSF group. # *p* < 0.05, ## *p* < 0.01, ### *p* < 0.001, #### *p* < 0.0001 M-DCSF group vs M-SCSF group. ^ *p* < 0.05, ^^ *p* < 0.01, ^^^ *p* < 0.001, ^^^^ *p* < 0.0001H-DCSF group vs H-SCSF group.

Traditionally, the effects of steaming on the ‘dryness-like’ properties of CSF were based on empirical experience rather than scientific evaluation. Therefore, here we conducted a systematic phenotypic analysis on rats to verified whether they truly had “dry-like” effects and the differences between them. Water intake was one of the core physiological indicators for evaluating the “dryness-like” response in rats. From a modern physiological perspective, when the body was exposed to “dryness-inducing” substances, which led to conditions such as fluid imbalance and mucosal dryness, it triggered changes in drinking behavior through neuroregulatory mechanisms. Therefore, monitoring water intake could directly reflect the intensity of the “dryness-like” effect (Zhu et al., 2020). The effects of DCSF and SCSF on water intake in rats were shown in Fig. 4A. Compared with the blank control group, all dosage groups of both DCSF and SCSF significantly increased water consumption, with the DCSF groups, especially at high doses, which exhibited a notably stronger effect than the corresponding SCSF groups. This result suggested that the “dryness-like” effect, as reflected by increased water intake, was more pronounced in DCSF than in its steamed counterpart.

Whole-blood viscosity is a key indicator reflecting blood fluidity, and its level was directly related to the smoothness of blood circulation. The fluid imbalance caused by “dryness-inducing” substances might lead to abnormal viscosity, which affected blood rheological properties. Therefore, measuring whole-blood viscosity at different shear rates could reveal the differences in “dryness-like” properties from the perspective of hemorheology (Tabesh et al., 2022). To further explore the specific mechanisms underlying the differences in “dryness-like” properties between DCSF tea and SCSF tea, we measured whole-blood viscosity at various shear rates (Fig. 4B). Both DCSF and SCSF treatments led to significant, dose-dependent increased in blood viscosity, and this effect was consistently greater for DCSF across low, medium, and high shear rates. These findings indicated that steaming processing alters functional properties related to the rheological aspects of the sample, which potentially reflected compositional changes.

Aquaporins (AQPs) are critical for water transport and homeostasis (Ke et al., 2025). AQP2 (renal water reabsorption), AQP3 (colon/kidney water absorption), and AQP5 (submandibular gland saliva secretion) are key to “dryness-like” responses, regulating fluid balance in tissues linked to dryness manifestations (dry mouth, altered urine, intestinal moisture). We focused on these three isoforms due to their tissue-specific roles and measured their mRNA expression to explore molecular mechanisms of differential “dryness-like” effects. To this end, we analyzed the mRNA expression of these aquaporins (AQP2, AQP3, and AQP5), key regulators of water transport and homeostasis in submandibular gland, kidney and colon tissues (Fig. 4C-E). Both DCSF and SCSF were found to downregulated AQP2 and AQP5 and upregulated AQP3 in a dose-dependent manner, with DCSF exerting consistently stronger effects at each dose than SCSF. These trends might reflect changes in bioactive components that influence in vivo water regulation mechanisms. Collectively, the results revealed that steaming substantially modified the compositional and functional characteristics of DCSF, which led to an attenuated “dryness-like” effect as demonstrated by in vivo physiological indicators and associated gene expression. Therefore, we conducted a more detailed analysis of the composition of the administered extracts.

### 3.6 Active components analysis

**Fig. 5:**
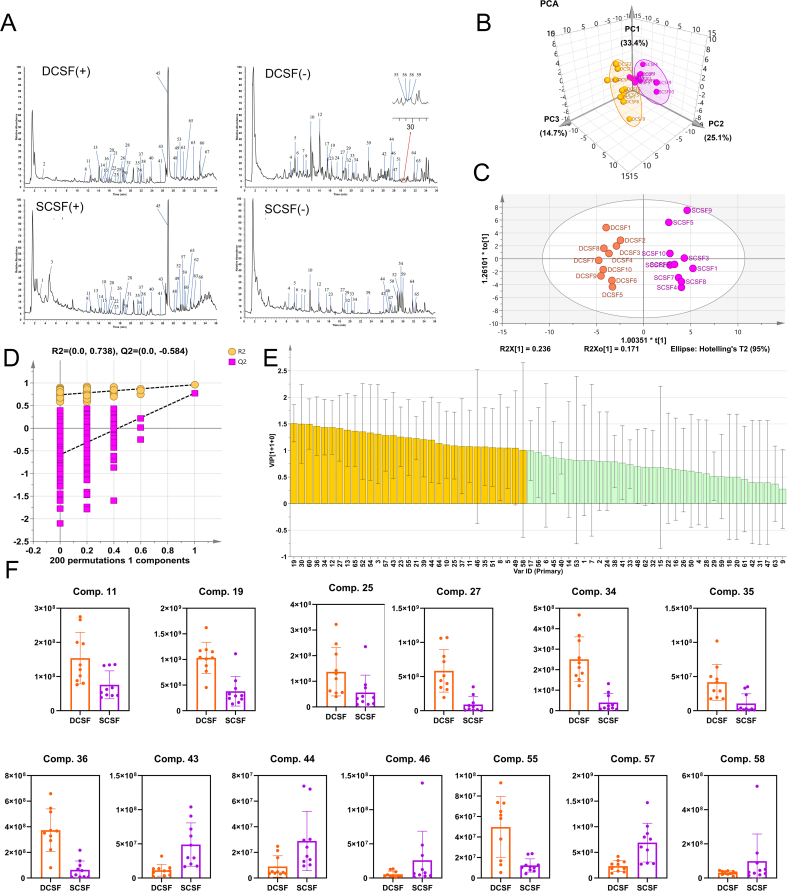
Analysis of active components identified by LC-MS. (A) Positive and negative ion TIC (Total Ion Current) ion chromatograms of the identified active components metabolites of DCSF vs SCSF; (B) PCA score scatter plot composed of all active components metabolites of DCSF vs SCSF; (C—D) OPLS-DA (Orthogonal Partial Least Squares Discriminant Analysis) model established by using active components metabolites as variables and DCSF vs SCSF as classes; (E) VIP (Variable Importance in Projection) of the established OPLS-DA model; (F) The change of flavonoids and their glycosides VIP > 1 in DCSF vs SCSF.

The gavage solutions were prepared from two types of tea, namely DCSF and SCSF. The inherent compositional differences between DCSF and SCSF drove the variations in their biological effects. Consequently, we performed a detailed comparative analysis of their gavage solutions to elucidate the compositions of these two gavage solutions with UHPLC-MS/MS. Through such an analysis, we aimd to identify the differences in their components, and further correlated these chemical changes with the actually observed biological effects such as dryness, thereby to gain a deeper understanding of the material basis underlying the generation of dryness. The TICs of the metabolic maps of DCSF and SCSF were shown in Fig. 5A. To confirm the structures of the metabolites, we compared them with standards and references (Deng et al., 2023; Zheng et al., 2022). A total of 67 chemical components were identified in DCSF and SCSF, including 27 flavonoids and their glycosides, 18 coumarins, 4 limonoids, 5 organic acids, 3 glycosides, 1 nucleoside and its base, 1 amino acid, 1 anthraquinone, and 7 other types. Flavonoids and their glycosides were the main chemical components in DCSF tea and SCSF tea (Table S5). Unbiased principal component discriminant analysis (PCA) confirmed that the metabolic components of DCSF and SCSF were indeed different (Fig. 5B) and could be well divided into two categories, indicating that the active chemical components had changed after steaming.

Given that DCSF and SCSF exhibited distinct drying properties, we hypothesized that the differential components between them might have been the key factors responsible for such variations. To verify this, we aimed to explore the differences in their ‘dryness-like’ effects” by establishing an orthogonal partial least-squares discriminant model (OPLS-DA) to compare the differential components before and after the steaming of DCSF (Fig. 5C). The Q value of the model is 0.80, indicating that the established OPLS-DA model was reliable and could accurately identify differential metabolites. In the fitting model diagram (Fig. 5D), R2Y (yellow dots) was above Q2Y (pink dots), indicating that the model was not over-fitted, had a strong explanatory ability for the dependent variable, and also reflects the adaptability of the data characteristics to the model. Therefore, we further analyzed the differential components of DCSF and SCSF through the VIP value. As shown in Fig. 5E, a total of 31 components had VIP> 1. We further analyzed these 31 important differential components (Fig. 5F; Fig. S5). Among them, there are 13 flavonoids and their glycosides, 7 coumarins, 2 organic acids, 2 glycosides, 2 limonoids, and 5 other types. Flavonoids and their glycosides accounted for about 50% of the total.

Therefore, we focused on these flavonoids and their glycoside compounds: isovitexin-2”-O-arabinoside (component 19), rhamnetin 3-rutinoside (component 36), kaempferol-3-O-rutinoside (component 34), rutin (component 27), cyanidin (component 43), 5,3′,4′-trihydroxy-6,7,8-trimethoxyflavone (component 55), 3-methoxyapigenin (component 57), luteolin (component 44), isovitexin (component 25), vicenin II (component 11), neohesperidin (component 35), 3-*O*-methylquercetin (component 58), quercetin (component 46). The study found that the contents of these components all changed significantly after steaming. The content of flavonoid glycoside components decreased significantly after steaming, while the content of flavonoid aglycone components increased significantly after steaming. Therefore, we speculated that flavonoid glycosides were converted into flavonoid aglycones. In the process of the cleavage of flavonoid glycosides into flavonoid aglycones, the glycosidic bond was the first chemical bond to break. The glycosidic bond is a covalent bond that connected the flavonoid aglycone and the sugar molecule. Under high-temperature conditions, the stability of the glycosidic bond decreased, and it was prone to breakage. The breakage of the glycosidic bond caused the sugar molecule to detach from the flavonoid aglycone, thus forming the flavonoid aglycone (Hvattum & Ekeberg, 2003). For example, isovitexin was formed by the loss of a glucose unit from isovitexin-2”-O-arabinoside or vicenin II. The decrease in the content of isovitexin-2”-O-arabinoside was precisely due to the above-mentioned process. Similarly, rutin may have lost a glucose molecule during the steaming process to form quercetin or isoquercitrin. The same principle applied to the conversion from naringin to naringenin and from kaempferol-3-O-rutinoside to kaempferol. 5,3′,4′-Trihydroxy-6,7,8-trimethoxyflavone was converted into 3-methoxyapigenin under demethylation conditions.

**Fig. 6:**
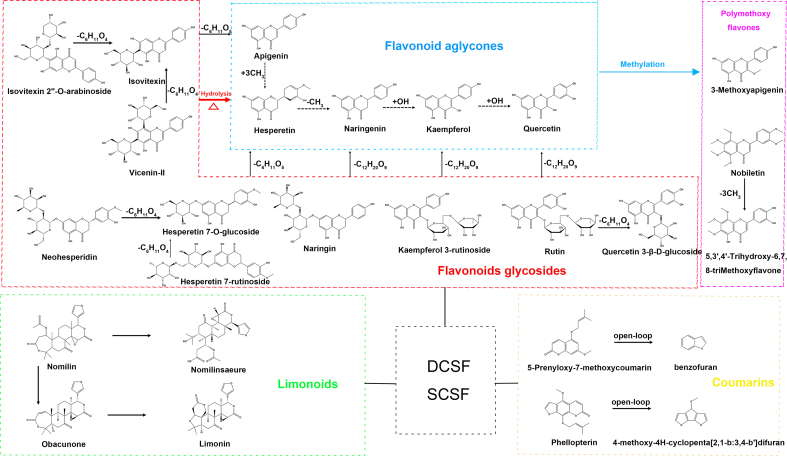
Possible conversion regularity of main active components metabolites between DCSF vs SCSF.

The contents of coumarin components also changed after steaming. The contents of components esculetin (component 8), 6,8-dihydroxy-7-methoxy-3-methylisocoumarin (component 30), 5,7-dihydroxy-4-methylcoumarin (component 37), bergapten (component 49) increased after steaming, while the contents of components 7-acetoxy-4-methylcoumarin (component 21), 5-prenoxy-7-methoxycoumarin (component 66), phellopterin (component 67) decreased after steaming. This is due to the ring-opening decomposition of the lactone structure of coumarin under high-temperature conditions. The limonoid components underwent internal transformation at high temperatures. Nomilin was converted into obacunone or nomilinic acid, and obacunone was converted into limonin, resulting in an increased in the contents of limonin, obacunone, and nomilinic acid and a decrease in the content of nomilin. The detailed process of structural transformation of these components was shown in Fig. 6.

It had been reported that flavonoid components, including rhoifolin, naringin, naringenin, poncirin, and eriodictyol-7-O-glucoside, had significant correlations with and contributions to the expression of renal AQP2, colonic AQP3, and submandibular gland AQP5, and served as the main drying components in *Fructus Aurantii* (Zhu et al., 2023). Additionally, such flavonoids could induce oral astringency, which in turn leads to xerostomia (dry mouth) (Liu et al., 2022). Rutin, as a typical flavonol component, had its astringency-inducing mechanism thoroughly studied: when rutin bound to salivary proteins in the oral cavity, it formed complexes. This binding not only consumed salivary proteins that played a lubricating role but also might generate insoluble precipitates, thereby increasing the frictional sensation in the oral cavity (Ferrer-Gallego et al., 2016). Therefore, we speculated that flavonoid glycosides and aglycone components were the material basis for the reduction of “dryness-like” effect. The decreased in the content of flavonoid glycoside components and the increased in the content of aglycones to achieve “steaming to reduce dryness-like effect “ were reasonable.

### 3.7 Molecular docking

**Fig. 7:**
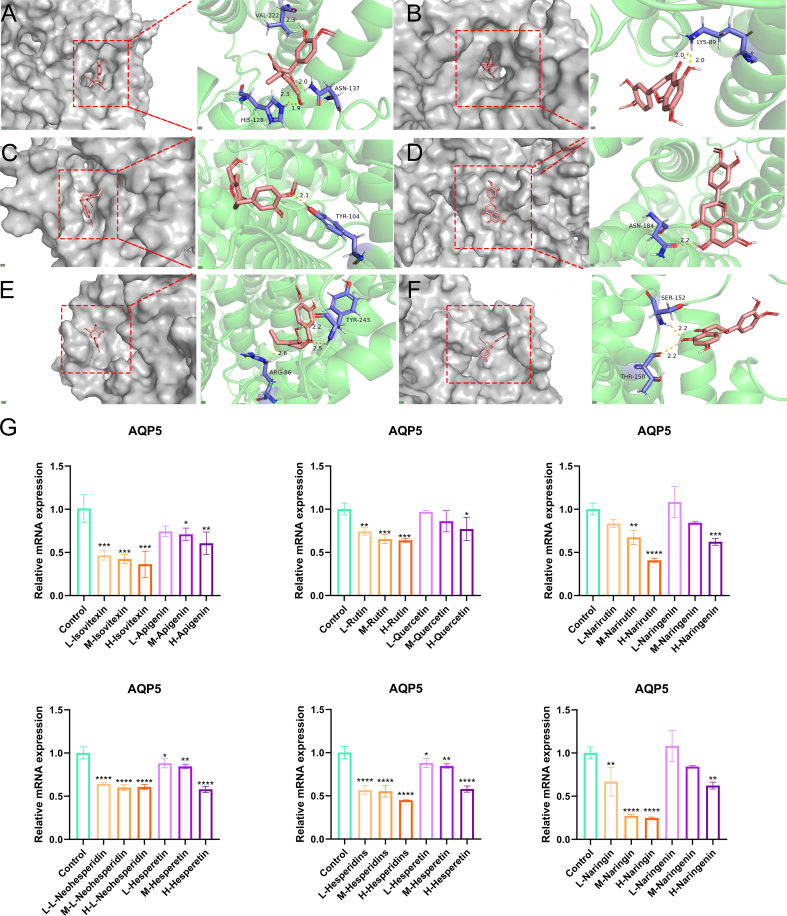
Molecular docking simulations and in vitro drying effect of flavonoid glycosides and flavonoid aglycones. (A) Docking simulation results of hesperidins with Aquaporin 2; (B) Docking simulation results of hesperetin with Aquaporin 2; (C) Docking simulation results of hesperidins with Aquaporin 3; (D) Docking simulation results of hesperetin with Aquaporin 3; (E) Docking simulation results of hesperidins with Aquaporin 5; (F) Docking simulation results of hesperetin with Aquaporin 5; (G) Effects of flavonoid glycosides and flavonoid aglycones on the mRNA expression level of aquaporin 5 (AQP5) in rat submandibular gland. * *p* < 0.05, ** *p* < 0.01, *** *p* < 0.001, **** *p* < 0.0001 vs Blank control group.

As documented in the literature, variations in substituents of anthocyanins—representative flavonoids—exerted a notable influence on astringency perception: glycosylated anthocyanins elicited a more intense astringent sensation, whereas their acetylated and cinnamoylated counterparts demonstrated a marked reduction in astringency (Paissoni et al., 2018). Similar structural modifications might also have impacted their interactions with other key targets involved in water metabolism. We hypothesized that the structural changes of flavonoids before and after steaming were correlated with their binding capacity to AQP proteins, which are associated with dryness properties. To this end, molecular docking studies were performed to compare the interactions between representative flavonoid glycosides and aglycones (identified in Section 3.6) with AQP proteins, and the results were presented in Fig. 7A-F and Table S6, which illustrated the binding modes and key interaction parameters, respectively. Docking simulations indicated that flavonoid aglycones, such as naringenin and quercetin, had significantly lower binding energies to AQP isoforms compared with their corresponding glycosides (naringin, rutin). For instance, the binding energy of quercetin to AQP2 was −9.76 kcal/mol, substantially lower than that of rutin (−8.25 kcal/mol), suggesting a higher binding affinity for the aglycone form. This enhanced binding was likely attributable to the smaller molecular size and reduced steric hindrance of aglycones compared to glycosides, which increased their fit within the AQP binding pocket (Sousa et al., 2006).

Further analysis showed that, despite forming fewer hydrogen bonds, aglycones interacted with key residues critical for AQP function, such as the involvement of Arg-188 in Apigenin-AQP3 binding. In contrast, glycosides, although forming more hydrogen bonds, exhibited weaker overall binding due to the peripheral position or low contribution of these interactions. Additionally, variations in the position and number of substituents on the flavonoid scaffold, especially multiple hydroxyl groups, were found to influence AQP subtype selectivity and binding energy (Guedes, Guedes et al., 2018). Collectively, these findings demonstrated that structurally modified flavonoid derivatives exhibit differential binding profiles to AQPs, a phenomenon that aligned with our hypothesis whereby steaming-induced structural alterations in flavonoids were associated with their interactive capacity with dryness-related AQP proteins.

### 3.8 Verification of “dryness-like” effect

While molecular docking had revealed binding differences between flavonoid glycosides and aglycones to AQPs, as well as the impact of structural modifications, such in silico simulation results remain simplified models that could not fully reflect the influence of complex factors in biological systems on actual functions. To verify whether these observed binding differences translated into dryness-related biological effects, studies at the cellular level, which are closer to physiological conditions, were needed to directly link structure-activity relationships with functional outcomes such as AQP expression regulation. Thus, we conducted single-component cellular experiments to validate the functions of these molecular interactions.

Specifically, we assessed the impact of these compounds on the expression of the AQP5 in NCI-H292 epithelial cells. Cell viability was first evaluated using the MTT assay after 24-h incubation with various concentrations (25, 50, 100 μg/mL) of selected flavonoid glycosides (hesperidin, neohesperidin, naringin, narirutin, rutin, isovitexin) and aglycones (hesperetin, naringenin, apigenin, quercetin). All compounds exhibited low cytotoxicity at these concentrations, with cell viability maintained above 80% (Fig. S6). Cells were treated with the same concentrations of flavonoid glycosides or aglycones for 4 h before quantification of AQP5 mRNA expression using qPCR (Fig. 7G). Both classes of compounds significantly downregulated AQP5 expression compared to the control. Notably, at every concentration tested, glycosides exhibited a more pronounced inhibitory effect on AQP5 expression than their corresponding aglycones. These results were statistically significant and consistent across all compound pairs examined. Collectively, these in vitro findings, combined with the in silico and in vivo data, suggested a potential mechanism where the steaming-induced conversion of flavonoid glycosides to aglycones alleviated the suppression of AQP5 expression. This compositional shift correlated with the attenuated “dryness-like” effects observed in SCSF, providing a plausible biochemical basis for the processing outcome observed in our animal models.

Recent studies had elucidated the core mechanism underlying the relationship between flavonoids, aquaporins (AQPs), and the dryness-like effect (Sun et al., 2025; Zhu et al., 2023). On the one hand, flavonoids could activate the PI3K-AKT signaling pathway to further regulate the specific expression of AQPs, and simultaneously synergize with other pathways (JAK-STAT3) to collectively modulate body water metabolism, inflammatory responses, and cyclic nucleotide balance, ultimately inducing a series of dryness symptoms including xerostomia, dry stool, and reduced salivary secretion (Zhu et al., 2023). On the other hand, the key to flavonoid-mediated alleviation of dryness resided in structural modification: deglycosylated or low-glycosylated flavonoids exhibited lower binding energy with AQPs, which directly restored the water transport activity of AQPs or downregulated abnormally overactivated signaling pathways, thereby restoring fluid metabolism function and ameliorating symptoms such as xerostomia (Sun et al., 2025). Furthermore, the observation that steaming could attenuate the dryness-like effect was consistent with the findings of a recent study (Lan et al., 2025).

Naturally, the present study had certain limitations. While we clarified the association between steaming-mediated flavonoid deglycosylation and enhanced AQP binding specificity, we did not perform in-depth validation of whether flavonoids modulate AQP expression and activity via specific receptors or signaling pathways (the specific regulatory nodes of cAMP and PI3K/Akt).

## 4 Conclusion

CSF formed its distinct quality through the critical processing step of steaming. The results demonstrated an association between steaming and the quality regulation of CSF via the alteration of its chemical composition. Steaming led to the reduction of fresh citrus volatiles and the increase of woody and bitter compounds, and promoted the conversion of flavonoid glycosides to aglycones as well as rearrangements of coumarins and limonoids. These chemical changes were observed to correlate with the enhancement of aroma and the alleviation of “dryness-like” effects.

This study went beyond traditional research focusing only on simple quality differences by establishing a framework connecting steaming with chemical and functional changes. These findings provided a scientific basis and plausible mechanism for standardizing CSF steaming processing and offered new strategies for quality regulation of similar medicinal-edible products. The combination of chemical changes and functional improvements provided practical insights for the standardized development of the CSF industry, with significance for both basic research and industrial applications.

## CRediT authorship contribution statement

**Xinhang Cai:** Writing – original draft, Validation, Supervision, Formal analysis, Data curation, Conceptualization. **Lishan Chen:** Formal analysis. **Wangjun Li:** Investigation. **Hu Wang:** Data curation. **Yue Sun:** Methodology. **Xingyang Xue:** Software. **Shumei Wang:** Data curation. **Menghua Wu:** Resources. **Jiang Meng:** Writing – review & editing, Funding acquisition.

## Declaration of competing interest

The authors declare that they have no known competing financial interests or personal relationships that could have appeared to influence the work reported in this paper.

## Data Availability

Data will be made available on request.

## Appendix A Supplementary data

Supplementary material Image 1
