# Supplementary material for: Effects of steam processing conditions on the aroma and “dryness-like” effect of Citri Sarcodactylis fructus(FoShou tea)
Source: Food Chem X. 2026 Feb 1;34:103590. doi: 10.1016/j.fochx.2026.103590 (PMC12887779; doi:10.1016/j.fochx.2026.103590)
Supplement: Supplementary file 1 — Supplementary material [file mmc1.docx]

**Supplementary Material**

**Effects of Steam Processing Conditions on the Aroma and "Dryness-like" effect of *Citri Sarcodactylis Fructus*(FoShou tea)**

Xinhang Cai^a,b^, Lishan Chen^a,b^, Wangjun Li^b^, Hu Wang^b^, Yue Sun^b^, Xingyang Xue^c*^, Shumei Wang^b*^, Menghua Wu^d,*^, Jiang Meng^a,b**^

^a^Department of Gastroenterology, The First Affiliated Hospital of Guangdong Pharmaceutical University, Guangzhou, Guangdong, China

^b^College of Traditional Chinese Medicine, Guangdong Pharmaceutical University/State Administration of Traditional Chinese Medicine Key Laboratory of Digital Quality Evaluation of Chinese Medicinal Materials/Guangdong Provincial General Colleges and Universities Engineering and Technology Research Center for Quality of Chinese Medicinal Materials, Guangzhou, China

^c^ Affiliated Cancer Hospital of Guangzhou Medical University, Guangzhou, China

^d^ College of Pharmacy, Jinan University, Guangzhou, China

^**^ Corresponding author: Department of Traditional Chinese Medicine, Guangdong Pharmaceutical University, Guangzhou 510006, China.

^*^ Corresponding authors: E-mail addresses: 2395903468@qq.com (S. Wang), 2439939086@qq.com (X. Xue), zyfxwmh@163.com (M. Wu), jiangmeng666@126.com (J. Meng).

**Contents**

**1:** LC-MS Gradient Elution Conditions

**2:** HS**-**GC-MS Programmed Temperature Conditions

**3:** Electronic Nose Sensor Information

**4:** Animal RT-qPCR Amplification Conditions and Primer Sequences for Aquaporins Detection

**5:** Cell RT-qPCR Amplification Conditions and Primer Sequences for Aquaporins Detection

**Figure S1:**Fingerprint chromatogram of common peaks from HS-GC-MS methodological validation

**Figure S2:**Total Ion Chromatogram of HS-GC-MS for Citri Sarcodactylis Fructus

**Figure S3:** Total Ion Chromatogram of HS-GC-MS for Steamed Citri Sarcodactylis Fructus

**Figure S4:** Results of 200 permutation tests for the three PLSR models

**Figure S5:** Analysis of active components identified by LC-MS

**Figure S6:** Characterization of in vitro cell viability

**Table S1:** Details of sample information

**Table S2:** Details of experimental materials

**Table S3:** Methodological Validation Results

**Table S4:** HS-GC-MS analysis between the *volatile* compounds of Citri Sarcodactylis Fructus with/without steaming processing

**Table S5:** Identification of the active components metabolites of Citri Sarcodactylis Fructus with/without steaming processing by using UHPLC-Q Exactive-Orbitrap-MS

**Table S6:** The optimal binding energy values and amino acid residues involved in interactions

**1.LC-MS Gradient Elution Conditions**

Mobile phase acetonitrile (A)-0.1% formic acid water (B), gradient elution: 0-1 min, 2%A; 1-4 min, 2-8%A; 4-10 min, 8-17%A; 10-15 min, 17-20%A; 15-20 min, 20-25%A; 20-22 min, 25-32%A; 22-27 min, 32-47%A; 27-33 min, 47-80%A; 33-34 min; 80-2%A; 34-36 min; 2%A;
**2. HS-GC-MS Programmed Temperature Conditions**

The temperature programming conditions were as follows: the column temperature was initially held at 60 °C for 1 min, then increased to 110 °C at a rate of 4 °C/min and held for 0 min, followed by a ramp to 130 °C at 10 °C/min and maintained for 5 min, followed by a ramp to 140 °C at 2 °C/min and maintained for 2 min, followed by a ramp to 190 °C at 5 °C/min and maintained for 0 min, and finally increased to 250 °C at 10 °C/min and maintained for 3 min.

**3. Electronic Nose Sensor Information**

W1C (aromatic organic compounds), W5S (nitrogen oxides), W3C (ammonia and aromatic substances), W6S (hydrogen), W5C (alkanes, aromatics, and nonpolar compounds), W1S (methane and various organic compounds), W1W (sulfides), W2S (ethanol and certain aromatic compounds), W2W (aromatic compounds and sulfides), and W3S (high concentrations of methane and aliphatic compounds).

**4. Animal RT-qPCR Amplification Conditions and Primer Sequences for Aquaporins Detection**

PCR amplification conditions: 95 ℃, 30 s predenaturation; 95 ℃, 5 s, 60 ℃, 30 s, lasting 40 cycles. The relative expression of aquaporins in each tissue of rats was calculated using *β*-actin as the internal reference. The primer sequence is as follows: AQP2-Forward/ AQP2-Reverse：GAGAAAGAGAGAGGGAGGGAGGAAG/ ACTGCTCGGGAGAAGGCTATGG； AQP3-Forward/ AQP3-Reverse：CTGTGGTTCCGTGGCTCAAGTG/ GATGGCAAGGGTGACAGCGAAG； AQP5-Forward/ AQP5-Reverse：CACAACTATGCCGCTGAACAACAAC/ AGAGTCGGTGGAGGAGAAGATGC； *β*-actin -Forward/ *β*-actin -Reverse：GCTCTCTTCCAGCCTTCCTT/ GGTCTTTACGGATGTCAACG；

**5. Cell RT-qPCR Amplification Conditions and Primer Sequences for Aquaporins Detection**

cDNA:The reaction program was as follows: 95 °C for 30 s, 1 cycle; 95 °C for 5 s, 60 °C for 30 s, 40 cycles; and then held at 4 °C.

mRNA:The reaction program was: 95 °C for 30 s, 1 cycle; 95 °C for 5 s, 62 °C for 20 s, 40 cycles; 95 °C for 1 h; 55 °C for 30 s; 95 °C for 30 s.

The gene primer sequences were as follows: *β*-actin -Forward/ *β*-actin -Reverse: GGACTTCGAGCAAGAGATGG/ AGCACTGTGTTGGCGTACAG; AQP5-Forward/ AQP5-Reverse: CGGGCTTTCTTCTACGTGG/ GCTGGAAGGTCAGAATCAGTC.


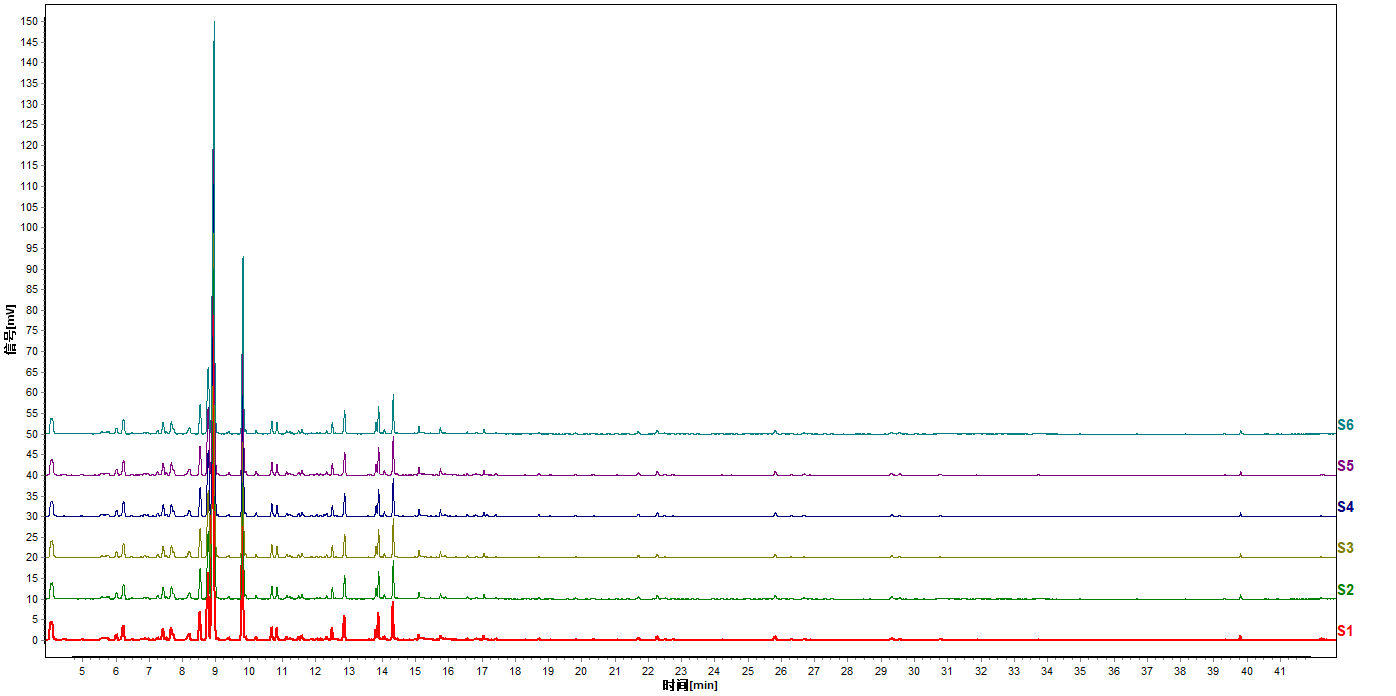


**Figure S1** Fingerprint chromatogram of common peaks from HS-GC-MS methodological validation


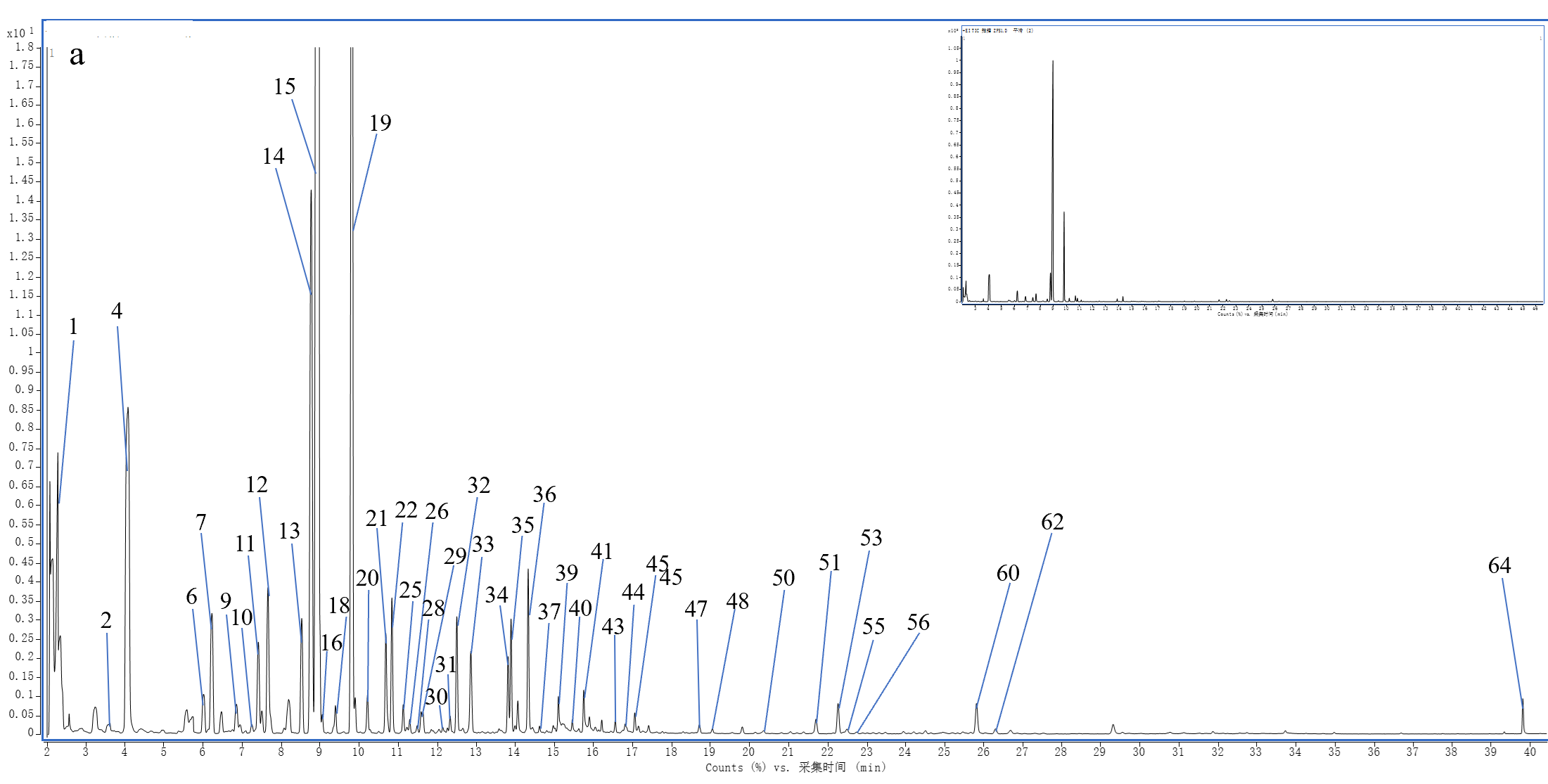


**Figure S2** Total Ion Chromatogram of HS-GC-MS for DCSF


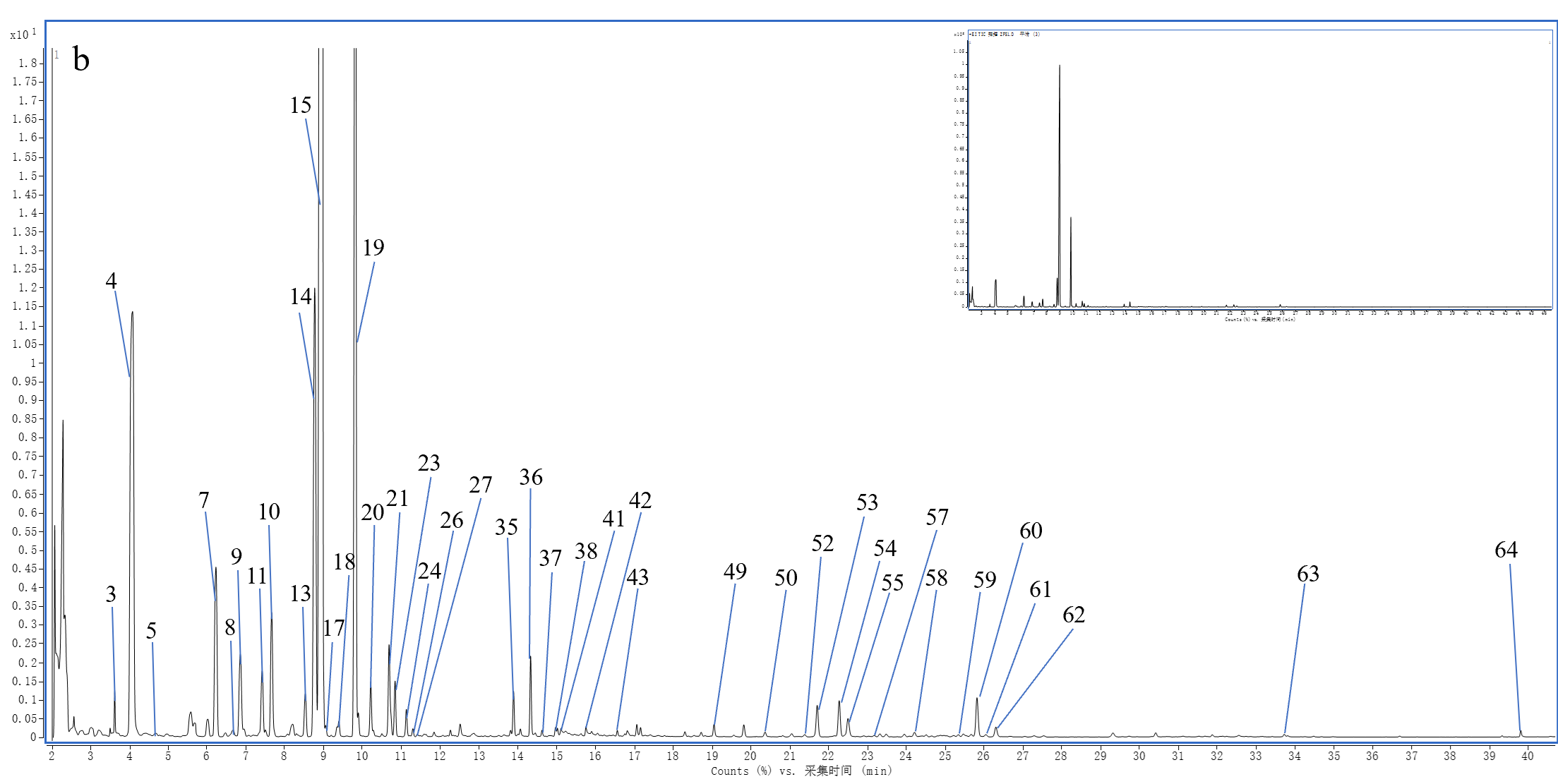


**Figure S3** Total Ion Chromatogram of HS-GC-MS for SCSF


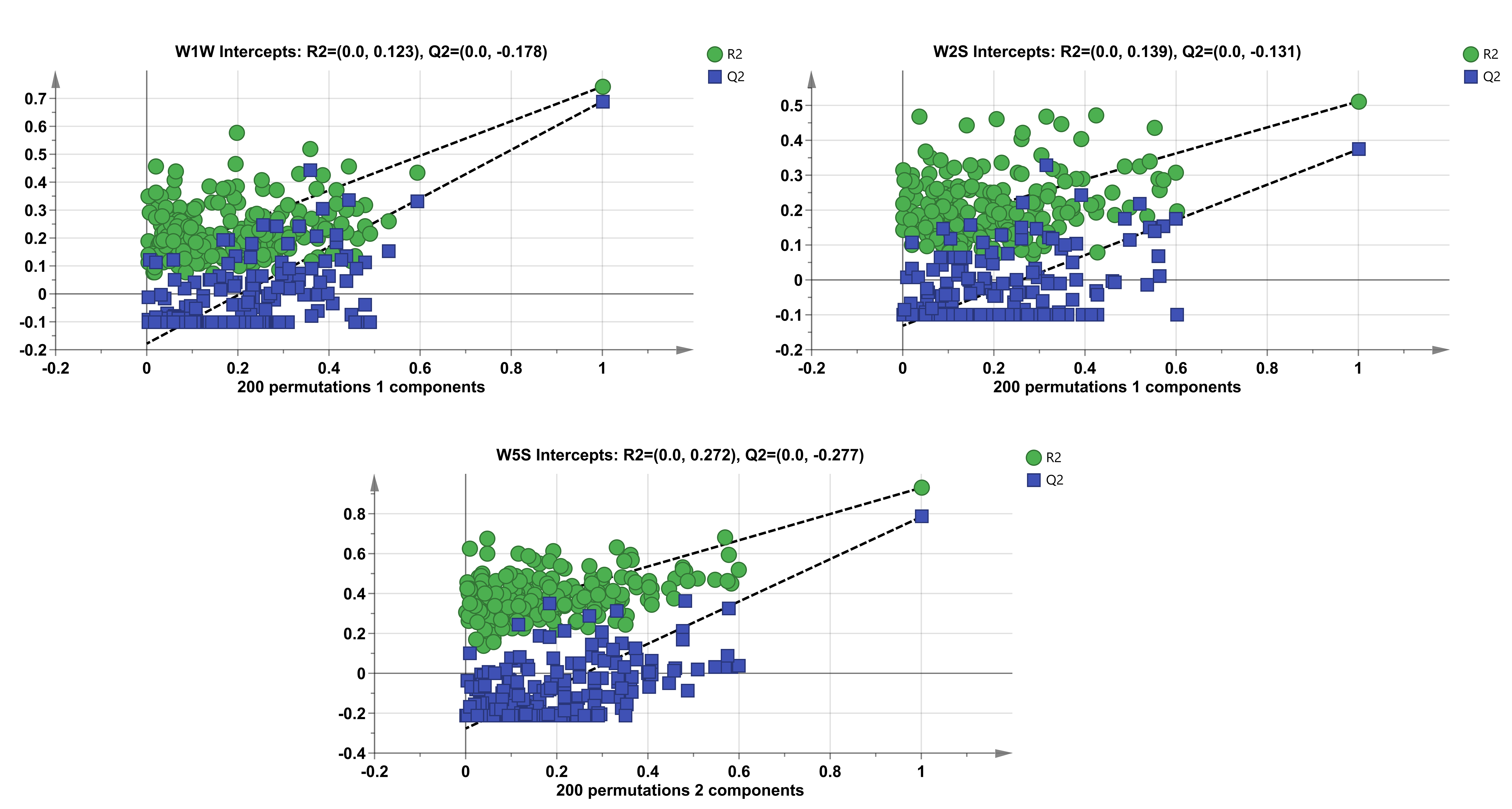


**Figure S4.** Results of 200 permutation tests for the three PLSR models


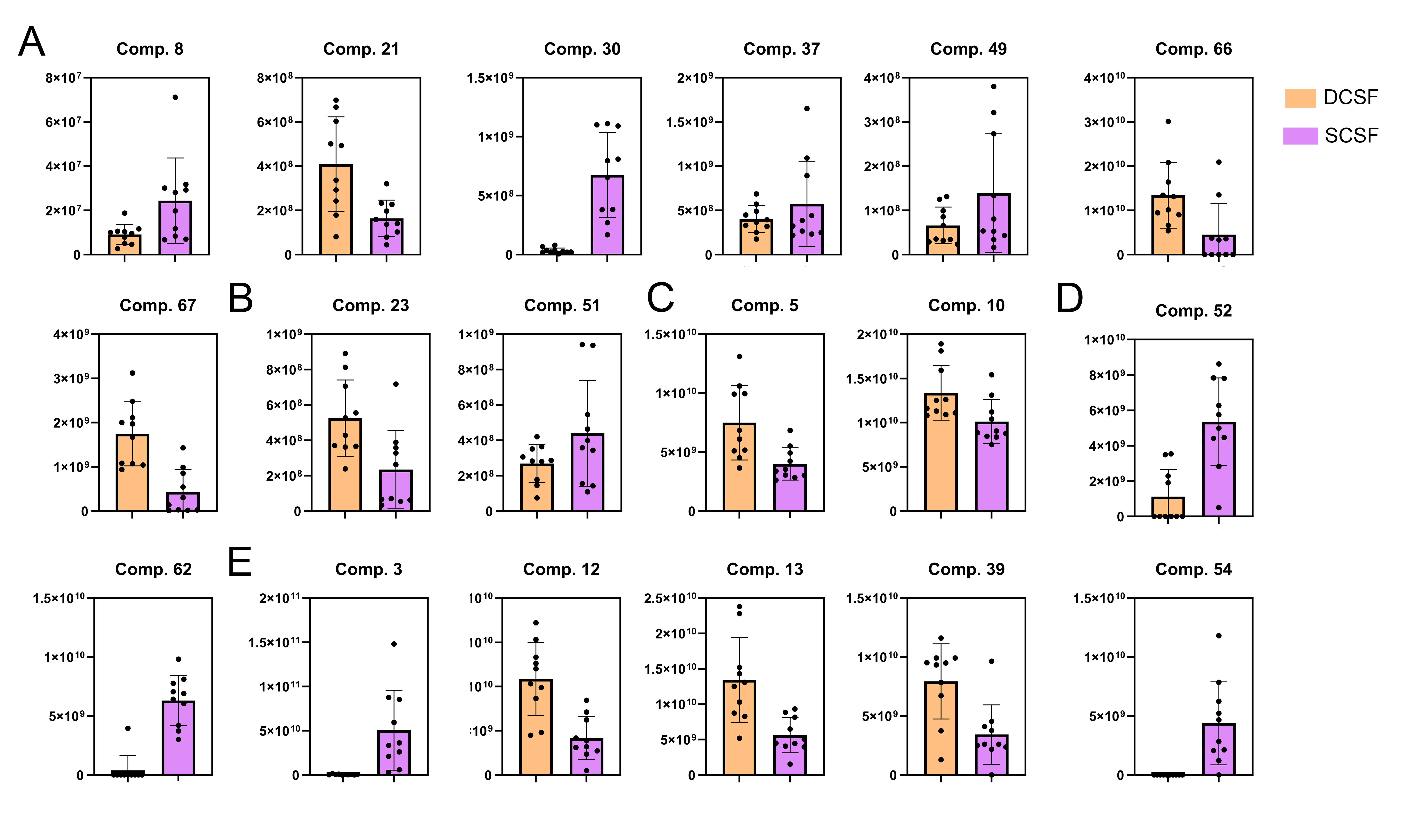


**Figure S5.** Analysis of active components identified by LC-MS. (A) The change of Coumarin compounds VIP > 1 in directly dried and then sliced Citri Sarcodactylis Fructus(DCSF) vs steaming processing then dried and sliced Citri Sarcodactylis Fructus(SCSF); (B) The change of Organic acids VIP > 1 in DCSF vs SCSF; (C) The change of Glycosides VIP > 1 in DCSF vs SCSF; (D) The change of Limonoids VIP > 1 in DCSF vs SCSF; (E) The change of Other categories VIP > 1 in DCSF vs SCSF.


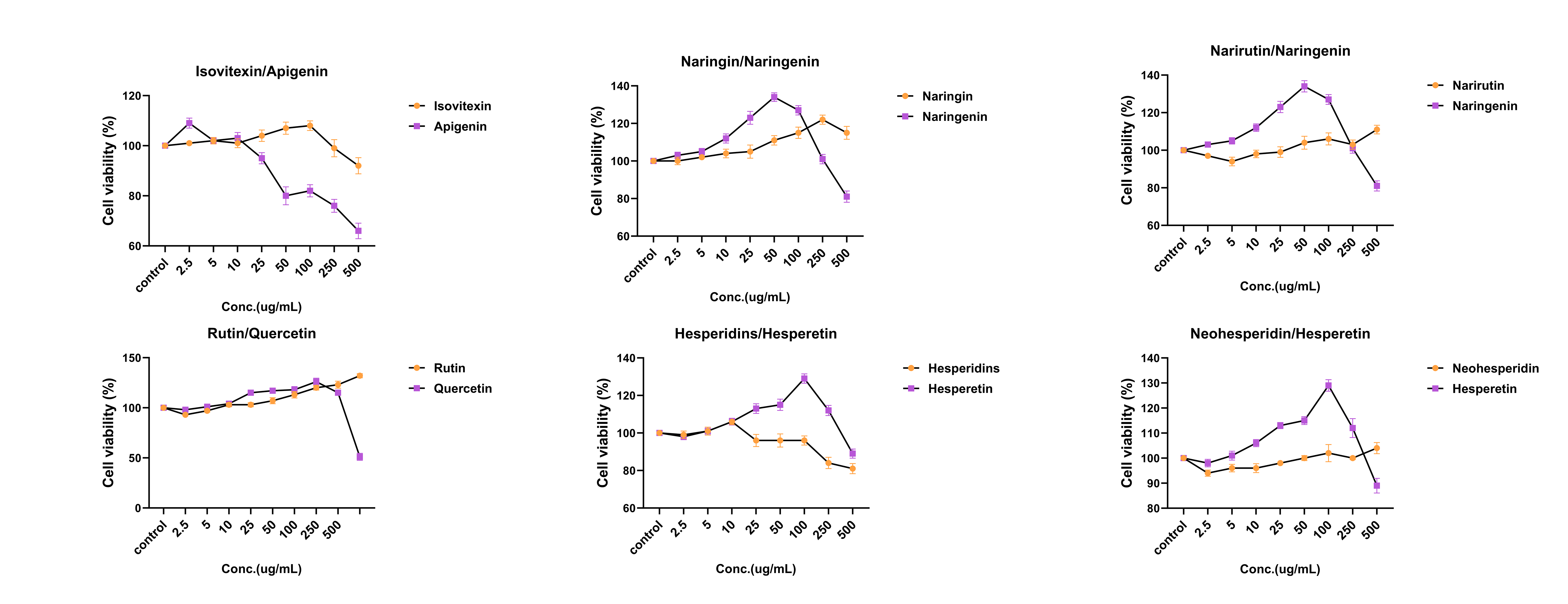
**Figure S6.** Characterization of in vitro cell viability. Cell viability at different times after the addition of different concentrations of flavonoid glycosides and flavonoid aglycones.

**Table S1.** Details of sample information

| No. | Company | Batch Number | Origin | Appearance |
| --- | --- | --- | --- | --- |
| DCSF 1 | Guangzhou Caizhilin Pharmaceutical Co., Ltd. | YPB0K0001 | Guangdong | Light yellow flake |
| DCSF 2 | Guangzhou Caizhilin Pharmaceutical Co., Ltd. | YPB0I0001 | Guangdong | Light yellow flake |
| DCSF 3 | Kangmei Pharmaceutical Co., Ltd. | 200604311 | Guangdong | Light yellow flake |
| DCSF 4 | Guangdong Shizhen Pharmaceutical Co., Ltd. | 201201 | Guangdong | Light yellow flake |
| DCSF 5 | Guangdong Daxiang Pharmaceutical Co., Ltd. | WLY21K01 | Guangdong | Light yellow flake |
| DCSF 6 | Kangmei Pharmaceutical Co., Ltd. | 210700971 | Guangdong | Light yellow flake |
| DCSF 7 | Kangmei Pharmaceutical Co., Ltd. | 230604021 | Guangdong | Light yellow flake |
| DCSF 8 | Sinopharm Group Fengliaoxing (Foshan) Medicinal Materials and Decoction Pieces Co., Ltd. | C22111057 | Guangdong | Light yellow flake |
| DCSF 9 | Zhixin Chinese Herbal Pieces Co., Ltd. | 211129 | Guangdong | Light yellow filament |
| DCSF 10 | Guangdong Hexiang Pharmaceutical Co., Ltd. | HX22R01 | Guangdong | Light yellow filament |
| SCSF 1 | Guangzhou Caizhilin Pharmaceutical Co., Ltd. | YPB0K0001 | Guangdong | Brownish-black flake |
| SCSF 2 | Guangzhou Caizhilin Pharmaceutical Co., Ltd. | YPA9D0001 | Guangdong | Brownish-black flake |
| SCSF 3 | Kangmei Pharmaceutical Co., Ltd. | 191202951 | Guangdong | Brownish-black flake |
| SCSF 4 | Guangdong Guangdong Junbang Pharmaceutical Co., Ltd. | 201101 | Guangdong | Brownish-black flake |
| SCSF 5 | Zhixin Chinese Herbal Pieces Co., Ltd. | 210201 | Guangdong | Brownish-black flake |
| SCSF 6 | Zhixin Chinese Herbal Pieces Co., Ltd. | HX21R01 | Guangdong | Brownish-black flake |
| SCSF 7 | Weida Chinese Herbal Pieces Co., Ltd. | 210901 - 1 | Guangdong | Brownish-black flake |
| SCSF 8 | Guangzhou Caizhilin Traditional Chinese Medicine Clinic | 210801 | Guangdong | Brownish-black flake |
| SCSF 9 | Sinopharm Group Fengliaoxing (Foshan) Medicinal Materials and Decoction Pieces Co., Ltd. | C22111057 | Guangdong | Brownish-black flake |
| SCSF 10 | Kangmei Pharmaceutical Co., Ltd. | 230600051 | Guangdong | Brownish-black flake |

**Table S2.** Details of experimental materials

| Category | Reagent Name | Batch Number | Company |
| --- | --- | --- | --- |
| Standard Compounds | 5-Hydroxymethylfurfural | CHB201120 | Chengdu Keloma Biological Company |
|  | Scopoletin | CHB201202 | Chengdu Keloma Biological Company |
|  | 6,7-Dimethoxycoumarin | CHB201102 | Chengdu Keloma Biological Company |
|  | Hesperetin | CHB180524 | Chengdu Keloma Biological Company |
|  | Isovitexin | CHB-Y-105 | Chengdu Keloma Biological Company |
|  | Apigenin | CHB201124 | Chengdu Keloma Biological Company |
|  | Naringenin | CHB180916 | Chengdu Keloma Biological Company |
|  | Rutin | AZ21080201 | Chengdu Keloma Biological Company |
|  | Naringin | CHB201202 | Chengdu Keloma Biological Company |
|  | Narirutin | CHB201208 | Chengdu Keloma Biological Company |
|  | Nobiletin | CHB210107 | Chengdu Keloma Biological Company |
|  | Luteolin | CHB201228 | Chengdu Keloma Biological Company |
|  | Hesperidin | RFS-C00601910011 | Chengdu Ruifensi Biological Technology Co., Ltd. |
|  | 5,7-Dimethoxycoumarin | RFS-E02801903022 | Chengdu Ruifensi Biological Technology Co., Ltd. |
|  | Bergapten | RFS-F00811811006 | Chengdu Ruifensi Biological Technology Co., Ltd. |
|  | Neohesperidin | X-010-190812 | Chengdu Ruifensi Biological Technology Co., Ltd. |
|  | 7-Hydroxycoumarin | RFS-S02511812016 | Chengdu Ruifensi Biological Technology Co., Ltd. |
|  | Quercetin | RFS-H00911805016 | Chengdu Ruifensi Biological Technology Co., Ltd. |
|  | Ferulic Acid | RFS-A00211812016 | Chengdu Ruifensi Biological Technology Co., Ltd. |
| Cell Culture | RPMI-1640 Medium | 8122664 | Gibco Company |
|  | Fetal Bovine Serum | SA22019 | Gibco Company |
|  | 0.25% Trypsin | 2444393 | Gibco Company |
|  | Penicillin/Streptomycin (Double Antibody) | 2441839 | Gibco Company |
|  | Dimethyl Sulfoxide (DMSO) | Y191207 | MP Biomedicals Company |
|  | PBS Phosphate Buffer Solution | 122033 | Meilunbio Company |
|  | 3-(4,5-Dimethylthiazol-2-yl)-2,5-Diphenyltetrazolium Bromide (MTT) | D1225A | Meilunbio Company |
| Molecular Biology | AG RNAex Pro RNA Extraction Kit | AG21102 | Hunan Accurate Biotechnology Co., Ltd. |
|  | Evo M-MLV Reverse Transcription Kit | AG11728 | Hunan Accurate Biotechnology Co., Ltd. |
|  | SYBR Premix Ex Taq HS Premixed qPCR Kit | AG11701 | Hunan Accurate Biotechnology Co., Ltd. |
| Other Reagents | Formic Acid | 220622 | Fisher Scientific, USA |
|  | Methanol | 220424 | Fisher Scientific, USA |
|  | Acetonitrile | 219087 | Fisher Scientific, USA |
|  | Experimental Water | 20220628 | Watsons, CHN |

Unless otherwise specified, all chemical reagents used in this study were of analytical grade.

**Table S3.** Methodological Validation Results (n=6; RSD %)

| LC-MS | | | | | HS-GC-MS | | | | | | | | Electronic Nose | | | |
| --- | --- | --- | --- | --- | --- | --- | --- | --- | --- | --- | --- | --- | --- | --- | --- | --- |
| Component | Precision | | Repeatability | Stability | Peak | Repeatability | Peak | Repeatability | Peak | Repeatability | Peak | Repeatability | Sensor | Repeatability | Sensor | Repeatability |
| 5-HMF | | 2.85 | 1.19 | 2.39 | Peak1 | 4.29 | Peak8 | 3.42 | Peak15 | 4.39 | Peak22 | 4.49 | W1C | 0.78 | W2S | 1.36 |
| Scopoletin | | 3.56 | 2.23 | 3.61 | Peak2 | 2.28 | Peak9 | 3.27 | Peak16 | 4.67 | Peak23 | 4.36 | W5S | 1.04 | W2W | 2.86 |
| 6,7-Dimethoxycoumarin | | 2.01 | 1.34 | 1.19 | Peak3 | 3.61 | Peak10 | 2.77 | Peak17 | 3.7 |  |  | W3C | 0.43 | W3S | 0.27 |
| Hesperidin | | 4.87 | 3.67 | 2.57 | Peak4 | 3.87 | Peak11 | 3.13 | Peak18 | 3.62 |  |  | W6S | 0.19 |  |  |
| 5,7-Dimethoxycoumarin | | 2.06 | 0.70 | 0.85 | Peak5 | 4.83 | Peak12 | 4.55 | Peak19 | 3.44 |  |  | W5C | 0.26 |  |  |
| Hesperetin | | 1.88 | 3.14 | 3.74 | Peak6 | 3.06 | Peak13 | 3.6 | Peak20 | 4.13 |  |  | W1S | 0.32 |  |  |
| Bergapten | | 1.16 | 1.78 | 1.42 | Peak7 | 3.84 | Peak14 | 4.73 | Peak21 | 3.31 |  |  | W1W | 1.39 |  |  |

**Table S4.** HS-GC-MS analysis between the volatile compounds of Citri Sarcodactylis Fructus with/without steaming processing.

| **No.** | **RT (min)** | **Identification** | **Molecular formula** | **Molecular weight** | **CAS** | **Percentage(%)** | |
| --- | --- | --- | --- | --- | --- | --- | --- |
|  |  |  |  |  |  | **CSF** | **SCSF** |
| 1 | 2.143 | 2-methyl-3-buten-2-ol | C_5_H_10_O | 86.13 | 115-18-4 | 1.6890±0.6143 | 0.0000±0.0000 |
| 2 | 3.61 | Hexanal | C_6_H_12_O | 100.16 | 66-25-1 | 0.3190±0.4247 | 0.0000±0.0000 |
| 3 | 3.618 | (+)-2,3 - butanediol | C_4_H_10_O_2_ | 90.12 | 19132-06-0 | 0.0000±0.0000 | 0.3190±0.3012 |
| 4 | 4.071 | 3-Furaldehyde | C_5_H_4_O_2_ | 96.08 | 498-60-2 | 4.3220±1.4608 | 13.9370±5.2023 |
| 5 | 4.682 | *α*-Angelica lactone | C_5_H_6_O_2_ | 98.10 | 591-12-8 | 0.0000±0.0000 | 0.0340±0.0227 |
| 6 | 6.004 | *α*-Phellandrene | C_10_H_16_ | 136.23 | 99-83-2 | 0.5840±0.2727 | 0.0000±0.0000 |
| 7 | 6.22 | *α*-Thujene | C_10_H_16_ | 136.23 | 2867-05-2 | 1.8200±0.9390 | 2.7650±1.1348 |
| 8 | 6.667 | Santolina triene | C_10_H_16_ | 136.23 | 2153-66-4 | 0.0000±0.0000 | 0.1010±0.0363 |
| 9 | 6.855 | 5-methylfuraldehyde | C_6_H_6_O_2_ | 110.11 | 620-02-0 | 0.2360±0.0810 | 1.3490±0.4012 |
| 10 | 7.247 | Bicyclo[3.1.0]hex-2-ene, 4-methyl-1-(1-methylethyl)- | C_10_H_16_ | 136.23 | 28634-89-1 | 0.1250±0.1082 | 1.4240±0.2018 |
| 11 | 7.409 | *β*-terpinene | C_10_H_16_ | 136.23 | 99-84-3 | 1.1710±0.6147 | 0.8760±0.3754 |
| 12 | 7.66 | (−)-*β*-pinene | C_10_H_16_ | 136.23 | 18172-67-3 | 1.7210±0.3215 | 0.0000±0.0000 |
| 13 | 8.524 | *α*-terpinene | C_10_H_16_ | 136.23 | 99-86-5 | 1.4240±0.7975 | 0.5030±0.1583 |
| 14 | 8.768 | *p*-Cymene | C_10_H_14_ | 134.22 | 99-87-6 | 6.8240±1.2945 | 6.3220±1.0075 |
| 15 | 8.955 | *D*-Limonene | C_10_H_16_ | 136.23 | 5989-27-5 | 52.2440±6.6030 | 54.0110±6.7733 |
| 16 | 9.048 | *trans*-*β*-Ocimene | C_10_H_16_ | 136.23 | 3779-61-1 | 0.1310±0.0536 | 0.0000±0.0000 |
| 17 | 9.052 | Toluene | C_10_H_16_ | 136.23 | 108-88-3 | 0.0000±0.0000 | 0.1680±0.0697 |
| 18 | 9.387 | Bicyclo[3.1.1]hept-2-ene, 3,6,6-trimethyl- | C_10_H_16_ | 136.23 | 4889-83-2 | 0.2080±0.0573 | 0.2400±0.1281 |
| 19 | 9.813 | 3-Carene | C_10_H_16_ | 136.23 | 13466-78-9 | 14.4990±3.6611 | 16.7880±3.9485 |
| 20 | 10.209 | *trans*-Linalool Oxide | C_10_H_18_O_2_ | 170.25 | 34995-77-2 | 0.2390±0.1404 | 0.2930±0.1474 |
| 21 | 10.678 | (+)-4-Carene | C_10_H_16_ | 136.23 | 13837-63-3 | 0.8440±0.3061 | 1.0750±0.1886 |
| 22 | 10.833 | 4-Methyl-*trans*-*β*-methylstyrene | C_10_H_12_ | 132.20 | 2077-30-7 | 0.7480±0.3506 | 0.0000±0.0000 |
| 23 | 10.835 | 4-ethenyl-1,2-dimethylbenzene | C_10_H_12_ | 132.20 | 27831-13-6 | 0.0000±0.0000 | 0.3550±0.1663 |
| 24 | 11.122 | Linalool | C_10_H_18_O | 154.25 | 78-70-6 | 0.0000±0.0000 | 0.1880±0.0529 |
| 25 | 11.126 | Linalyl acetate | C_12_H_20_O_2_ | 196.29 | 115-95-7 | 0.2220±0.0581 | 0.0000±0.0000 |
| 26 | 11.29 | Nonanal | C_9_H_18_O | 142.24 | 124-19-6 | 0.0550±0.0178 | 0.0400±0.0067 |
| 27 | 11.402 | Maltol | C_6_H_6_O_3_ | 126.11 | 118-71-8 | 0.0000±0.0000 | 0.0110±0.0032 |
| 28 | 11.481 | (2S,4R)-4-Methyl-2-(2-methylprop-1-en-1-yl)tetrahydro-2H-pyran | C_10_H_18_O | 154.25 | 3033-23-6 | 0.0530±0.0472 | 0.0000±0.0000 |
| 29 | 11.584 | (6,6-Dimethylbicyclo[3.1.1]hept-2-en-2-yl)methyl ethyl carbonate | C_13_H_20_O_3_ | 192.30 | 65861-93-0 | 0.2080±0.1037 | 0.0000±0.0000 |
| 30 | 12.139 | 2,6-Dimethyl-1,3,5,7-octatetraene, E,E- | C_10_H_14_ | 134.22 | 460-01-5 | 0.0350±0.0284 | 0.0000±0.0000 |
| 31 | 12.327 | *p*-Mentha-1,5,8-triene | C_10_H_14_ | 134.22 | 21195-59-5 | 0.1100±0.0616 | 0.0000±0.0000 |
| 32 | 12.5 | 1,3,8-*p*-Menthatriene | C_10_H_14_ | 134.22 | 18368-95-1 | 0.6690±0.3028 | 0.0000±0.0000 |
| 33 | 12.857 | Ethanone, 1-(1,4-dimethyl-3-cyclohexen-1-yl)- | C_10_H_16_O | 152.23 | 43219-68-7 | 0.7490±0.4038 | 0.0000±0.0000 |
| 34 | 13.806 | Citral | C_10_H_16_O | 152.23 | 5392-40-5 | 0.4820±0.2112 | 0.0000±0.0000 |
| 35 | 13.886 | 3-Cyclohexen-1-ol, 4-methyl-1-(1-methylethyl)-, (R)- | C_10_H_18_O | 154.25 | 20126-76-5 | 1.1480±0.4581 | 0.3750±0.142 |
| 36 | 14.325 | *α*-terpinyl formate | C_11_H_18_O_2_ | 182.26 | 2153-26-6 | 1.5460±0.6109 | 0.7370±0.1947 |
| 37 | 14.617 | 1-Tridecyne | C_13_H_24_ | 180.33 | 26186-02-7 | 0.0380±0.0042 | 0.0270±0.0067 |
| 38 | 14.962 | *p*-menth-1-en-9-al | C_10_H_16_O | 152.23 | 29548-14-9 | 0.0000±0.0000 | 0.0300±0.0141 |
| 39 | 15.099 | *cis*-Chrysanthenyl formate | C_11_H_16_O_2_ | 180.24 | 241123-18-2 | 0.3010±0.1287 | 0.0000±0.0000 |
| 40 | 15.456 | Neral | C_10_H_16_O | 152.23 | 106-26-3 | 0.0620±0.0305 | 0.0000±0.0000 |
| 41 | 15.748 | Nerol | C_10_H_18_O | 154.25 | 106-25-2 | 0.3220±0.0924 | 0.0410±0.0251 |
| 42 | 15.749 | Geraniol | C_10_H_18_O | 154.25 | 106-24-1 | 0.0000±0.0000 | 0.0500±0.02 |
| 43 | 16.553 | Thymol | C_10_H_14_O | 150.22 | 89-83-8 | 0.0760±0.0295 | 0.0250±0.0108 |
| 44 | 16.811 | Phenol, 2-methyl-5-(1-methylethyl)- | C_10_H_14_O | 150.22 | 499-75-2 | 0.0720±0.023 | 0.0000±0.0000 |
| 45 | 17.06 | 3-Methyl-4-isopropylphenol | C_10_H_14_O | 150.22 | 3228-02-2 | 0.1250±0.0479 | 0.0000±0.0000 |
| 46 | 17.411 | Ethanone, 1-(2-hydroxy-5-methylphenyl)- | C_9_H_10_O_2_ | 150.17 | 1450-72-2 | 0.0650±0.0172 | 0.0000±0.0000 |
| 47 | 18.71 | *cis*-muurola-3,5-diene | C_15_H_24_ | 204.35 | 157374-44-2 | 0.0750±0.0268 | 0.0000±0.0000 |
| 48 | 19.047 | 1,6-Octadien-3-ol, 3,7-dimethyl-, formate | C_11_H_18_O_2_ | 182.26 | 115-99-1 | 0.0490±0.0145 | 0.0000±0.0000 |
| 49 | 19.047 | Neryl acetate | C_12_H_20_O_2_ | 196.29 | 141-12-8 | 0.0000±0.0000 | 0.0990±0.0778 |
| 50 | 20.358 | 1,5-Cyclodecadiene, 1,5-dimethyl-8-(1-methylethenyl)-, [S-(Z,E)]- | C_15_H_24_ | 204.35 | 75023-40-4 | 0.0420±0.0114 | 0.0350±0.0118 |
| 51 | 21.695 | Bicyclo[5.2.0]nonane, 2-methylene-4,8,8-trimethyl-4-vinyl- | C_15_H_24_ | 204.35 | 1005245-76-0 | 0.2360±0.085 | 0.0000±0.0000 |
| 52 | 21.702 | (-)-isocaryophyllene | C_15_H_24_ | 204.35 | 118-65-0 | 0.0000±0.0000 | 0.7590±0.4810 |
| 53 | 22.265 | *cis*-*α*-Bergamotene | C_15_H_24_ | 204.35 | 18252-46-5 | 0.3500±0.0945 | 0.0340±0.0151 |
| 54 | 22.267 | *trans*-*α*-Bergamotene | C_15_H_24_ | 204.35 | 17699-05-7 | 0.0000±0.0000 | 0.7190±0.2859 |
| 55 | 22.487 | Ethanone, 1-(2-hydroxy-5-methoxyphenyl)- | C_9_H_10_O_3_ | 166.17 | 705-15-7 | 0.0000±0.0000 | 0.0860±0.0773 |
| 56 | 22.503 | Ethanone, 1-(2-hydroxy-4-methoxyphenyl)- | C_9_H_10_O_3_ | 166.17 | 552-41-0 | 0.1340±0.2422 | 0.0000±0.0000 |
| 57 | 23.316 | 1,5,9,9-Tetramethyl-1,4,7-cycloundecatriene | C_15_H_24_ | 204.35 | 515812-15-4 | 0.0000±0.0000 | 0.0480±0.0274 |
| 58 | 24.202 | Naphthalene, 1,2,3,4,4a,5,6,8a-octahydro-7-methyl-4-methylene-1-(1-methylethyl)-, (1*α*,4a*β*.,8a*α*)- | C_15_H_24_ | 204.35 | 39029-41-9 | 0.0000±0.0000 | 0.0820±0.0483 |
| 59 | 25.33 | *α*-Muurolene | C_15_H_24_ | 204.35 | 10208-80-7 | 0.0000±0.0000 | 0.0460±0.035 |
| 60 | 25.807 | 1H-Benzocycloheptene, 2,4a,5,6,7,8,9,9a-octahydro-3,5,5-trimethyl-9-methylene-, (4aS-cis)- | C_15_H_24_ | 204.35 | 3853-83-6 | 0.3920±0.1182 | 0.8220±0.3475 |
| 61 | 26.042 | (+)-*epi*-Bicyclosesquiphellandrene | C_15_H_24_ | 204.35 | 54274-73-6 | 0.0000±0.0000 | 0.0350±0.0222 |
| 62 | 26.297 | *δ*-cadinene | C_15_H_24_ | 204.35 | 483-76-1 | 0.0750±0.0347 | 0.2440±0.1613 |
| 63 | 33.718 | *α*-Bisabolol | C_15_H_26_O | 222.37 | 515-69-5 | 0.0000±0.0000 | 0.0230±0.0082 |
| 64 | 39.804 | Methyl palmitate | C_17_H_34_O_2_ | 270.45 | 112-39-0 | 0.2270±0.1623 | 0.1040±0.0490 |

**Table S5.** Identification of the active components metabolites of Citri Sarcodactylis Fructus with/without steaming processing by using UHPLC-Q Exactive-Orbitrap-MS.

|  | **RT**  **(min)** | **Selected ion** | **Formula** | **Measured mass** | **δ/ppm** | **MS/MS(m/z)** | **Name** |
| --- | --- | --- | --- | --- | --- | --- | --- |
| 1 | 2.63 | [M+H]^+1^ | C_1__0_H_13_N_5_O_4_ | 268.1040 | -0.14 | 268.1040、136.0618、137.0589 | Adenosine |
| 2 | 3.68 | [M+H]^+1^ | C_9_H_11_NO_2_ | 166.0864 | 0.49 | 120.0810、166.0864、121.0844、149.0598、103.0547 | Phenylalanine |
| 3 | 4.86 | [M+H]^+1^ | C_6_H_6_O_3_ | 127.0392 | 1.62 | 127.0392、109.0288、81.0342、53.0395 | 5-HMF |
| 4 | 8.52 | [M-H]^-1^ | C_15_H_16_O_9_ | 339.0723 | 0.38 | 339.0723、177.0185、133.0285 | Esculin |
| 5 | 9.31 | [M-H]^-1^ | C_15_H_26_O_10_ | 365.1451 | 3.003 | 365.145、303.1450、263.1137、221.1027、161.0447、125.0233、101.0231 | 1-O-(3-Butenyl)-6-O-alpha-L-arabinosyl-beta-D-glucopyranoside |
| 6 | 9.79 | [M-H]^-1^ | C_8_H_8_O_4_ | 167.0342 | -0.492 | 167.0342、123.0438、122.0362、95.0125 | 3,4-dihydroxyphenylacetic acid |
| 7 | 10.89 | [M-H]^-1^ | C_8_H_8_O_4_ | 167.0343 | -4.96 | 167.0343、123.0440、95.0126、81.0333、76.0176 | 1,3-Phenylenediacetic acid |
| 8 | 11.27 | [M+H]^+1^ | C_9_H_6_O_4_ | 179.0340 | 0.99 | 179.0340、151.0392、133.0286、123.0444 | Esculetine |
| 9 | 11.34 | [M-H]^-1^ | C_16_H_18_O_10_ | 369.0829 | 0.46 | 369.0829、207.0293、192.0256 | Fraxin |
| 10 | 12.30 | [M-H]^-1^ | C_16_H_22_O_9_ | 357.1191 | 3.168 | 195.0657、151.0755、136.0518 | Ethyl 3-methoxy-4-O-beta-D-glucosylbenzoate |
| 11 | 12.76 | [M+H]^+1^ | C_2__7_H_30_O_15_ | 595.1660 | -0.18 | 577.1559、541.1346、457.1133、427.1024 | Vicenin 2 |
|  | 12.31 | [M-H]^-1^ |  | 593.1513 | 0.47 | 593.1513、473.1087、353.0666、503.1198、575.1398 |  |
| 12 | 13.96 | [M-H]^-1^ | C_17_H_24_O_10_ | 387.1296 | 2.678 | 387.1296、225.0764、207.0657、181.0862、166.0626 | 3-epi-SwertiajaposideC |
| 13 | 13.98 | [M+H]^+1^ | C_11_H_14_O_5_ | 227.0912 | -0.749 | 227.0912、209.0808、181.0859、174.0764、167.0303、155.0703 | unknown |
| 14 | 14.00 | [M+H]^+1^ | C_10_H_8_O_4_ | 193.0498 | 0.32 | 193.0498、178.0263、165.0549、149.0599、133.0286 | Scopoletin |
| 15 | 14.19 | [M+H]^+1^ | C_10_H_8_O_4_ | 193.0496 | 0.61 | 193.0496、178.0262、165.0598、149.0598、133.0289 | 6,7-Dihydroxy-4-methylcoumarin |
| 16 | 14.99 | [M+H]^+1^ | C_9_H_6_O_3_ | 163.0390 | 0.47 | 163.0390、135.0441、119.0495、107.0496、91.0546 | 7-Hydroxycoumarin |
| 17 | 15.43 | [M-H]^-1^ | C_18_H_22_O_10_ | 397.1139 | 2.434 | 397.1139、235.0609、191.0707、176.0470 | 6-(beta-D-glucopyranosyloxy)-7-methoxy-5-benzofuranpropanoic acid |
| 18 | 15.56 | [M+H]^+1^ | C_27_H_32_O_15_ | 597.1815 | 0.04 | 596.1815、451.1237、425.1296、289.0706 | Neoeriocitrin |
| 19 | 15.74 | [M+H]^+1^ | C_26_H_28_O_14_ | 565.1549 | 0.15 | 433.1128、313.0705、283.0601、337.0705、415.1023 | Isovitexin 2''-O-arabinoside |
|  | 15.36 | [M-H]^-^^1^ |  | 563.1407 | 0.45 | 293.0455、413.0877、563.1407、323.0561 |  |
| 20 | 15.56 | [M+H]^+1^ | C_15_H_12_O_6_ | 289.0705 | -0.5 | 289.0705、271.0600、153.0183 | Eriodictyol |
| 21 | 15.84 | [M+H]^+1^ | C_12_H_10_O_4_ | 219.0652 | 0.13 | 177.0547、219.0652、165.0547、149.059 | 7-Acetoxy-4-methylcoumarin |
| 22 | 16.09 | [M+H]^+1^ | C_21_H_20_O_10_ | 433.1131 | 0.51 | 433.1131、415.1023、397.0919、367.0815、313.0707、283.0602 | Vitexin |
| 23 | 16.93 | [M+H]^+1^ | C_10_H_10_O_4_ | 195.0652 | 0.52 | 195.0652、177.0546、149.0598、145.0284、117.0338、93.0332 | Ferulic acid |
|  | 16.56 | [M-H]^-1^ |  | 193.0500 | -3.72 | 193.0500、178.0264、149.0597、134.0362 |  |
| 24 | 17.30 | [M-H]^-1^ | C_27_H_30_O_15_ | 593.1512 | 0.52 | 285.0403、447.0927、327.0510、286.0438 | Luteolin 7-O-rutinoside |
| 25 | 17.88 | [M+H]^+1^ | C_21_H_20_O_10_ | 433.1129 | 0.37 | 433.1129、415.1024、397.0917、367.8319、313.0707、283.0602 | Isovitexin |
| 26 | 17.90 | [M+H]^+^^1^ | C_11_H_10_O_4_ | 207.0654 | 0.65 | 192.0417、179.0704、163.0755、151.0755 | Scoparone |
| 27 | 18.60 | [M+H]^+1^ | C_27_H_30_O_16_ | 611.1604 | -0.05 | 465.1028、303.0499、129.0548、71.0499 | Rutin |
|  | 18.30 | [M-H]^-1^ |  | 609.1465 | 0.56 | 609.1465、610.1495、343.0458、301.0355、300.0274 |  |
| 28 | 18.43 | [M+H]^+1^ | C_27_H_32_O_14_ | 581.1867 | 0.3 | 273.0759、85.0291、195.0291、419.1337、129.0549 | Naringin |
| 29 | 18.57 | [M-H]^-1^ | C_9_H_16_O_4_ | 187.0969 | -3.76 | 187.0969、169.0862、125.0960、97.0646 | Azelaic Acid |
| 30 | 18.71 | [M+H]^+1^ | C_11_H_10_O_5_ | 223.0603 | 0.36 | 223.0603、193.0135、179.0705、163.0391 | 6,8-Dihydroxy-7-methoxy-3-methylisocoumarin |
| 31 | 18.83 | [M+H]^+1^ | C_28_H_34_O_15_ | 611.1973 | -0.25 | 303.0865、177.0549、153.0184、465.1394、431.1347、449.1441 | Hperidin |
| 32 | 19.53 | [M-H]^-1^ | C_21_H_20_O_12_ | 463.0887 | 0.76 | 300.0277、301.0358、271.0249、255.0296、178.9978、151.0028 | Isoquercetin |
| 33 | 20.15 | [M-H]^-1^ | C_28_H_32_O_15_ | 607.1668 | 1.752 | 607.1668、299.0560、284.0325 | Diosmin |
| 34 | 20.67 | [M-H]^-1^ | C_27_H_30_O_15_ | 593.1511 | 0.51 | 285.0407、284.0328、255.0298、257.0456 | Kaempferol-3-O-rutinoside |
| 35 | 21.14 | [M+H]^+1^ | C_28_H_34_O_15_ | 611.1972 | 0.58 | 303.0863、219.0288、177.0547、153.0185 | Neohesperidin |
| 36 | 21.15 | [M+H]^+1^ | C_28_H_32_O_16_ | 625.1765 | 0.04 | 491.7246、479.1186、317.0657、129.0548 | Narcissin |
| 37 | 21.79 | [M+H]^+1^ | C_10_H_8_O_4_ | 193.0496 | 0.29 | 193.0496、178.0262、165.0547、149.0598、133.0649 | 5,7-Dihydroxy-4-methylcoumarin |
| 38 | 22.41 | [M+H]^+1^ | C_10_H_8_O_3_ | 177.0546 | 0.33 | 177.0546、149.0599、133.0649、121.0651 | 4-Methylumbelliferone |
| 39 | 23.14 | [M-H]^-1^ | C_16_H_16_O_9_ | 351.0721 | 2.824 | 163.0391、177.0183、119.0489、92.1802 | unknown |
| 40 | 23.90 | [M+H]^+1^ | C_16_H_16_O_6_ | 305.1017 | -0.37 | 305.1017、203.0338、159.0439、147.0440、131.0492 | Oxypeucedanin hydrate |
| 41 | 25.49 | [M+H]^+1^ | C_12_H_12_O_5_ | 237.0758 | 0.05 | 237.0758、207.0289、191.0340、176.0470、222.0524 | 6,7,8-Trimethoxycoumarin |
| 42 | 26.08 | [M-H]^-1^ | C_19_H_24_O_5_ | 331.1555 | 0.45 | 331.1555、332.1588、287.1657、243.1029 | Gibberellin A3 |
| 43 | 26.49 | [M+H]^+1^ | C_18_H_16_O_8_ | 361.0918 | -0.01 | 361.0918、346.0683、328.0578、300.0629、272.0681 | Centaureidin |
| 44 | 27.27 | [M-H]^-1^ | C_15_H_10_O_6_ | 285.0405 | 0.47 | 285.0405、286.0438、241.0505、175.0395、151.0026、133.0284、199.0395 | Luteolin |
| 45 | 27.38 | [M+H]^+1^ | C_11_H_10_O_4_ | 207.0651 | -0.24 | 207.0651、192.0416、179.0702、163.0753、151.0753 | 5,7-Dimethoxycoumarin |
| 46 | 27.53 | [M-H]^-1^ | C_15_H_10_O_7_ | 301.0353 | -0.279 | 301.0353、178.9978、151.0026、121.0283 | Quercetin |
| 47 | 28.01 | [M-H]^-1^ | C_15_H_12_O_5_ | 271.0615 | 0.61 | 271.0615、253.0509、151.0027、119.0491、107.0126 | Naringenin |
| 48 | 28.02 | [M+H]^+1^ | C_16_H_14_O_6_ | 303.0863 | 0.14 | 303.0863、285.0761、177.0547、153.0183 | Hesperetin |
| 49 | 28.53 | [M+H]^+1^ | C_12_H_8_O_4_ | 217.0496 | 0.37 | 217.0496、202.0262、173.0598、161.0597、131.0492 | Bergapten |
| 51 | 28.93 | [M-H]^-1^ | C_16_H_30_O_4_ | 285.2072 | 0.16 | 285.2072、267.1967、241.2169、223.1706 | Hexadecanedioic acid |
| 50 | 28.72 | [M+HCOO]^-^ | C_26_H_30_O_8_ | 515.2277 | 2.109 | 469.1780、515.2277、229.1229、271.2279、161.0598 | Limonin |
|  |  | [M-H]^-1^ |  | 469.1780 |  |  |  |
| 52 | 29.19 | [M+H]^+1^ | C_28_H_36_O_10_ | 533.2380 | -0.144 | 4515.2277、469.2222、455.2061、411.2166、369.2060、161.0598 | Nomilinic acid |
|  | 29.22 | [M-H]^-1^ |  | 531.2235 | 1.951 | 531.2235、471.2025、427.2125、369.1715、245.1547 |  |
| 53 | 29.27 | [M+H]^+1^ | C_16_H_14_O_5_ | 287.2217 | -0.02 | 287.2217、203.0339、159.0443、147.0441 | Oxypeucedanin |
| 54 | 29.51 | [M-H]^-1^ | C_26_H_32_O_8_ | 471.2026 | 2.559 | 471.2026、427.2129、383.2229、325.1809、307.1706 | unknown |
| 55 | 29.54 | [M-H]^-1^ | C_18_H_16_O_8_ | 359.0775 | 0.88 | 359.0775、329.0304、344.0538、314.0069、301.0356、286.0119、258.0169、178.9977 | 5,3’,4’-Trihydroxy-6,7,8-trimethoxyflavone |
| 56 | 29.54 | [M-H]^-1^ | C_15_H_10_O_5_ | 269.0457 | 0.33 | 225.0555、151.0027、117.0333、107.0126 | Apigenin |
| 57 | 29.61 | [M+H]^+1^ | C_16_H_12_O_6_ | 301.0707 | 0.31 | 301.0707、286.0472、258.0522 | 3-Methoxyapigenin |
| 58 | 29.70 | [M-H]^-1^ | C_16_H_12_O_7_ | 315.0513 | 0.75 | 315.0511、300.0276、272.0328、271.0249、243.0297 | 3-O-Methylquercetin |
| 59 | 29.82 | [M+H]^+1^ | C_26_H_32_O_8_ | 473.2169 | -0.134 | 427.2116、409.2009、369.2006、341.2113、321.1848、161.0598、201.0910、99.0133 | unknown |
|  | 29.84 | [M-H]^-1^ |  | 471.2023 | 2.092 | 427.2121、383.2229、325.1809、307.1704 |  |
| 60 | 29.94 | [M+H]^+1^ | C_28_H_34_O_9_ | 515.2277 | -0.27 | 515.2277、469.2222、455.2065、411.2166、471.2372、369.2059、161.0598、 | Nomilin |
| 61 | 30.08 | [M+H]^+1^ | C_21_H_22_O_8_ | 403.1389 | 0.1 | 403.1389、388.1152、373.0918、355.0811、327.0864 | Nobiletin |
| 62 | 31.12 | [M+H]^+1^ | C_26_H_30_O_7_ | 455.2064 | -0.07 | 455.2064、437.1958、409.2009、391.1903、161.0598 | Obacunone |
| 63 | 31.67 | [M+H]^+1^ | C_18_H_16_O_7_ | 345.0967 | 0.13 | 345.0967、330.0731、346.0999 | Eupatilin |
| 64 | 31.69 | [M-H]^-1^ | C_17_H_14_O_7_ | 329.0667 | 0.06 | 314.0431、299.0195、271.0247、329.0665、243.0295 | Aurantio-obtusin |
| 65 | 30.25 | [M+H]^+1^ | C_19_H_18_O_8_ | 375.1074 | -0.07 | 375.1074、360.0839、345.0604、317.0655 | Casticin |
|  | 31.83 | [M-H]^-1^ |  | 373.0929 | 0.13 | 373.0929、358.0695、343.0459、315.0512、300.0277 |  |
| 66 | 32.83 | [M+H]^+1^ | C_15_H_16_O_4_ | 261.1121 | -0.174 | 261.1121、193.0497、165.0547、149.0598、137.0598、121.0649、109.0651 | 5-Prenyloxy-7-methoxycoumarin |
| 67 | 33.06 | [M+H]^+1^ | C_17_H_16_O_5_ | 301.1073 | 0.14 | 233.0447、218.0213、177.0549 | Phellopterin |

**Table S6.** The optimal binding energy values and amino acid residues involved in interactions.

| **Comp.** | **AQP2** | | **AQP3** | | **AQP5** | |
| --- | --- | --- | --- | --- | --- | --- |
|  | **Binding energy (kcal/mol)** | **Hydrogen Bond interactions** | **Binding energy (kcal/mol)** | **Hydrogen Bond interactions** | **Binding energy (kcal/mol)** | **Hydrogen Bond interactions** |
| Neohesperidin | -6.78 | Pro-99; Asp-100; Gly-105; Leu-104; Leu-103 | -6.21 | Arg-188; Asn-183 | -6.53 | Thr-150; Gln-81; Ser-64; Val-63 |
| Hesperidins | -6.32 | Val-222; Asn-137; His-128 | -6.33 | Tyr-104 | -6.85 | Tyr-243; Arg-86 |
| Hesperetin | -7.15 | Lys-89 | -7.79 | Asn-184 | -7.97 | Ser-152; Thr-150 |
| Narirutin | -7.86 | Asp-100 | -7.38 | Pro-102 | -7.95 | Arg-153; Ser-229 |
| Naringin | -7.63 | Asn-285; Gly-278; Ser-282 | -7.35 | Trp-98; Ile-99 | -7.04 | Val-158; Ser-149 |
| Naringenin | -9.27 | Asn-285; Ile-204 | -9.13 | Gln-24; Glu-28 | -9.08 | Ser-152; Leu-230 |
| Rutin | -8.25 | Gly-102; His-177; Leu-96 | -8.35 | Pro-181; Asn-184; Arg-188 | -8.99 | Gly-159; Asp-151 |
| Quercetin | -9.76 | Lys-234 | -9.67 | Asn-183; Arg-188 | -9.24 | Thr-155; Thr-150; Asp-151 |
| Isovitexin | -7.52 | Tyr-167; Leu-258 | -7.65 | Arg-20; His-16; Arg-18 | -7.12 | Glu-244 |
| Apigenin | -9.50 | Trp-192; Ala-195; Tyr-197 | -9.74 | Arg-188 | -9.19 | Lys-12; Leu-225 |
